# Structural Characterization and Anticoagulant Activities of a Keratan Sulfate-like Polysaccharide from the Sea Cucumber *Holothuria fuscopunctata*

**DOI:** 10.3390/md21120632

**Published:** 2023-12-08

**Authors:** Ru Chen, Weili Wang, Ronghua Yin, Ying Pan, Chen Xu, Na Gao, Xiaodong Luo, Jinhua Zhao

**Affiliations:** 1 State Key Laboratory of Phytochemistry and Plant Resources in West China, Kunming Institute of Botany, Chinese Academy of Sciences, Kunming 650201, China; chenru@mail.kib.ac.cn (R.C.); wangweili2303@163.com (W.W.); 2 Yunnan Institute of Traditional Chinese Medicine and Materia Medica, Kunming 650223, China; 3 University of Chinese Academy of Sciences, Beijing 100049, China; 4 School of Pharmaceutical Sciences, South-Central Minzu University, Wuhan 430074, China; yinrh77@163.com (R.Y.); panyingkkxx@outlook.com (Y.P.); 15171981226@163.com (C.X.); 5 Yunnan Characteristic Plant Extraction Laboratory, Key Laboratory of Medicinal Chemistry for Natural Resource, Ministry of Education and Yunnan Province, School of Chemical Science and Technology, Yunnan University, Kunming 650091, China

**Keywords:** aminoglycan, structural features, keratan sulfate, depolymerization, oligosaccharides, anticoagulant activity

## Abstract

A sulfated polysaccharide (AG) was extracted and isolated from the sea cucumber *H. fuscopunctata*, consisting of GlcNAc, GalNAc, Gal, Fuc and lacking any uronic acid residues. Importantly, several chemical depolymerization methods were used to elucidate the structure of the AG through a bottom-up strategy. A highly sulfated galactose (oAG-1) and two disaccharides labeled with 2,5-anhydro-D-mannose (oAG-2, oAG-3) were obtained from the deaminative depolymerized product along with the structures of the disaccharide derivatives (oAG-4~oAG-6) identified from the free radical depolymerized product, suggesting that the repeating building blocks in a natural AG should comprise the disaccharide β-D-GalS-1,4-D-GlcNAc_6S_. The possible disaccharide side chains (bAG-1) were obtained with mild acid hydrolysis. Thus, a natural AG may consist of a keratan sulfate-like (KS-like) glycosaminoglycan with diverse modifications, including the sulfation types of the Gal residue and the possible disaccharide branches α-D-GalNAc_4S6S_-1,2-α/β-L-Fuc_3S_ linked to the KS-like chain. Additionally, the anticoagulant activities of the AG and its depolymerized products (dAG1-9) were evaluated in vitro using normal human plasma. The AG could prolong activated partial thromboplastin time (APTT) in a dose-dependent manner, and the activity potency was positively related to the chain length. The AG and dAG1-dAG3 could prolong thrombin time (TT), while they had little effect on prothrombin time (PT). The results indicate that the AG could inhibit the intrinsic and common coagulation pathways.

## 1. Introduction

The body wall of sea cucumbers is rich in polysaccharides. Over the years, various polysaccharides from sea cucumbers have been analyzed, including sulfated fucan (SF), fucosylated chondroitin sulfate (FCS) or fucosylated glycosaminoglycan (FG), glucan and a novel sulfated polysaccharide composed of N-acetyl hexosamine, galactose and fucose. Particularly, the structures of FG and SF have been extensively investigated [1,2,3].

In general, the structure of the FG backbone is formed by alternating 3-linked D-GalNAc_4S6S_ and 4-linked D-GlcA units (-4-β-D-GlcA-1,3-β-D-GalNAc_4S6S_-1-), and the distinct sulfated fucosyl branches are usually attached at the O-3 position of the D-GlcA residue. Recently, the structural diversity of FG from different sea cucumber species has been elucidated, and the structural variants are usually revealed by their unique branches, especially a sulfation pattern, as well as their monosaccharide composition and number. For example, the structures differed in the sulfation types of FucS. Namely, most of the FucS in HfFG (FG from *Holothuria fuscopunctata*) was L-Fuc_3S4S_ [4], but the FucS in SvFG (FG from *Stichopus variegatus*) was L-Fuc_2S4S_ [5], while the FucS in HaFG (FG from *Holothuria albiventer*) was L-Fuc_3S4S_, L-Fuc_2S4S_ and L-Fuc_4S_ [6]. The unique disaccharide branches were confirmed as α-D-Gal_4S(6S)_-1,2-L-Fuc_3S_ with the structural elucidation of oligosaccharides prepared from *Thelenota ananas* [7].

Regarding the structure of SFs, SFs from sea cucumbers are usually composed of uniform L-fucose residues, but the glycosidic linkage and the sulfation pattern of these SFs may vary in different sea cucumber species. For example, the sulfated fucans consisting of distinctive simple repeating units have been isolated from *H. fuscopunctata*, *T. ananas* and *Stichopus horrens*, respectively [8]. The structural sequences of these sulfated fucans are [-4-α-L-Fuc_3S_-1-], [-4-α-L-Fuc_2S_-1-] and [-3-α-L-Fuc_2S_-1-], respectively. Meanwhile, several SFs are composed of 3-linked α-L-fucose units with 2- and/or 4- sulfation substitutions within repeating tetrasaccharide building blocks, for example, SFs from *Ludwigothurea grisea* [9], *Isostichopus badionotus* [10], *Acaudina molpadioides* [11], *Holothuria tubulosa* [12] and *Thelenota ananas* [13].

Additionally, neutral glucans have been found in several sea cucumber species [14,15], including *H. edulis* and *Pattalus mollis*.

Recently, four types of sulfated polysaccharides were investigated in the sea cucumber *H. fuscopunctata* [16], indicating the diversity of polysaccharides in the body wall of a sea cucumber species. A regular FG was identified through a bottom-up strategy [4], and the trisaccharide repeating unit is -4-[α-L-Fuc_3S4S_-1,3]-β-D-GlcA-1,3-β-D-GalNAc_4S6S_-1-. Particularly, two types of SFs, namely SFI and SFII, have been reported. The tetrasaccharide repeating units in SFI were elucidated as [-3-α-L-Fuc_2S4S_-1,3-α-L-Fuc_(2S)_-1,3-α-L-Fuc_2S_-1,3-α-L-Fuc_0S_-1-]_n_ based on the structure of a series of oligosaccharides released by mild acid hydrolysis [17]. By combining the application of the β-eliminative and deaminative depolymerization with glycosidic linkage cleavage selectivity, a highly regular SF (SFII) was found, and its structural sequence is [-4-α-L-Fuc_3S_-1-] [8,16]. Interestingly, a sulfated polysaccharide, composed of D-GlcNAc, D-GalNAc, D-Gal, L-Fuc, and no uronic acid residues was observed, so it was designated as aminoglycan (AG). However, the precise structure of the AG based on pure oligosaccharides has not been reported so far.

It is worth noting that keratan sulfate (KS) is a glycosaminoglycan (GAG) consisting of the repeating disaccharide unit -3-β-Gal-1,4-β-GlcNAc-1- [18,19]. Significantly, KS is the only GAG type that does not contain any uronic acid residues. In the disaccharide repeating unit of chondroitin sulfate, dermatan sulfate, hyaluronic acid and heparin/heparan sulfate, the glucuronic acid or iduronic acid could be observed. According to the monosaccharide compositions, the AG may be a KS-like polysaccharide found in sea cucumbers. However, its structure has not been elucidated.

Sulfated polysaccharides exhibit multiple biological activities [2], including anticoagulation and antithrombosis [1], anticancer, antivirus and immunomodulation. The anticoagulant activity and molecular mechanism of FCS have attracted extensive attention. In one of our recent reports, the natural AG showed moderate activated partial thromboplastin time (APTT) prolonging activity (5.84 IU/mg) compared to FCS with potent activity [16]. However, further work is needed to separate the oligosaccharides to investigate the structure–activity relationship of the AG.

In this study, we isolated and purified a natural AG from the sea cucumber *H. fuscopunctata*. The physicochemical properties and chemical composition of the AG were investigated. Importantly, the deacetylation and deaminative depolymerization was used to cleave the AG, and the resulting oligosaccharides were purified and structurally identified. The commonly used free radical depolymerization was applied to the AG to produce fragments of different molecular weights, which were used to explore the relationship between molecular size and biological activity. Additionally, three oligosaccharides were obtained and identified. Due to the presence of the fucose residues in the AG, the AG was further subjected to mild acid hydrolysis, and the released fragments were collected and analyzed. According to the structures of oligosaccharides, the tentative chemical structure of the AG was deduced. The anticoagulant activities of the natural AG and its related compounds were evaluated in vitro in human control plasma.

## 2. Results and Discussion

### 2.1. Structural Characterization of AG

**Figure 1:**
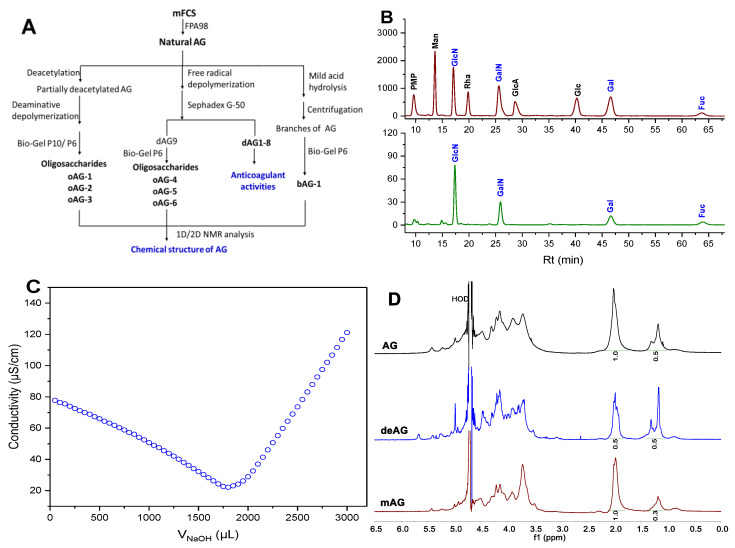
(**A**) Flow chart for the purification of the AG from the body wall of sea cucumber *H. fuscopunctata* and its related oligosaccharides oAG-1–6 and bAG-1. (**B**) Monosaccharide compositions of the AG. (**C**) Conductivity titration curve of the AG. (**D**) ^1^H NMR spectra of the AG, partially deacetylated AG (deAG) and the high-molecular-weight product of mild acid hydrolysis of the AG (mAG).

As shown in previous studies, the AG could be purified by a combination of β-eliminative depolymerization and strong anion exchange chromatography (Figure S1 and Figure 1A) [16]. The result of the monosaccharide composition analysis indicated that the homogeneous AG was composed of GlcN, GalN, Gal and Fuc (Figure 1B), which is consistent with a previous study [16]. The sulfate content was ~37% as determined through a conductivity titration method (Figure 1C) [14]. The ^1^H NMR spectrum of the AG is shown in Figure 1D, and the signals at the 2.01 ppm and 1.18 ppm with the integral ratio of 1:0.5 could be assigned to the methyl groups of two N-acetyl hexamines and Fuc, respectively. The 3.50–5.00 ppm region contains the signals of hydrogens in the sugar ring. The overlapping and broad signals in the ^1^H NMR spectrum of the AG were difficult to assign, indicating that the structure of the AG may be complex [16]. The approach applying specific degradation methods to cleave the polysaccharide chain to afford fragments has been shown to be effective in the structural elucidation of complex biomacromolecules [20]. Consequently, the natural AG was subjected to three chemical degradation treatments as shown in Figure 1A, and the released fragments were mainly characterized with 1D/2D NMR spectroscopy.

### 2.2. Preparation of Oligosaccharides with Deacetylation–Deaminative Depolymerization

Deaminative cleavage of amino sugar glycosides is an effective depolymerization approach to cleave the glycosidic linkages formed by hexosamine [21]. According to the reaction mechanism, this process could convert the N-deacylated amino sugars into 2,5-anhydrohexoses. As for the most common amino sugars in biopolymers, GlcN and GalN are converted to 2,5-anhydro-mannose (aMan) and 2,5-anhydro-talose (aTal), respectively. Thus, the oligosaccharide formed in the deamination reaction could be labeled with the 2,5-anhydrohexose terminal as the new reducing end. Regarding polysaccharides containing N-acetyl substituted amino sugar residues, the partial deacetylation procedure is required before deamination with acid [22].

As mentioned above, the AG was composed of four types of monosaccharides, including GlcNAc, GalNAc, Gal and Fuc. To elucidate the structure of the AG, its depolymerized product dAG was prepared with the method of deacetylation and deaminative depolymerization. After partial deacetylation through hydrazinolysis (deAG), the integral of the methyl signals assigned to the acetyl groups of the AG at 2.0 ppm was reduced (Figure 1D), compared with the natural AG, suggesting the formation of the unsubstituted amino groups.

**Figure 2:**
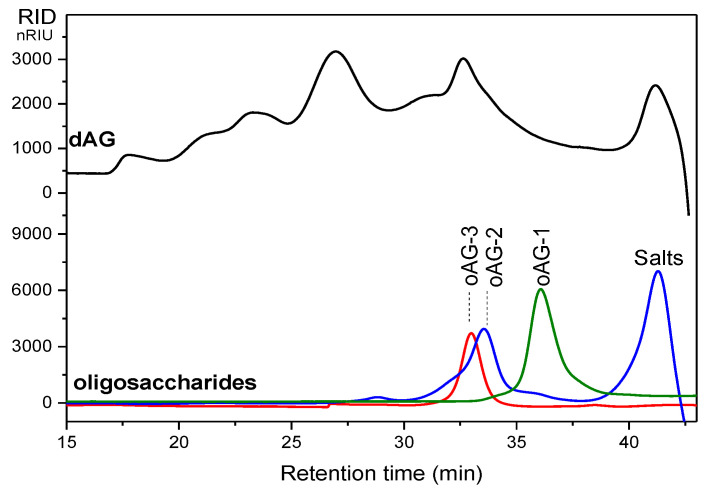
HPGPC profiles of dAG and oAG-1, oAG-2, oAG-3.

After deaminative depolymerization with nitrous acid at pH 4, the low-molecular-weight product composed of oligosaccharides was obtained. The high-performance gel permeation chromatography (HPGPC) profile of dAG analyzed on a Superdex Peptide 10/300 GL column showed a series of peaks with different retention times (Figure 2), indicating that dAG was composed of several oligosaccharides. After separation with GPC using Bio-gel P10 and P6 columns, three fractions oAG-1, oAG-2 and oAG-3 (Figure 2) were obtained, which were further subjected to 1D/2D NMR spectroscopy.

**Figure 3:**
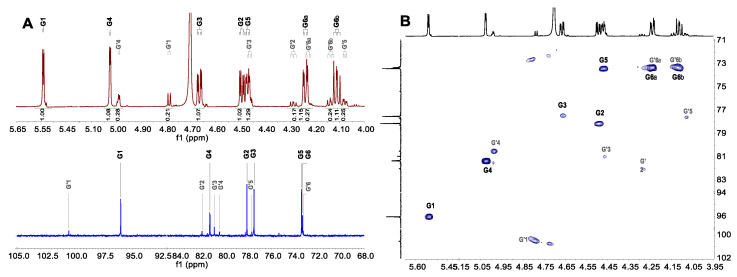
^1^H/^13^C NMR (**A**) and ^1^H-^13^C HSQC (**B**) spectra of oAG-1.

**Scheme 1:**
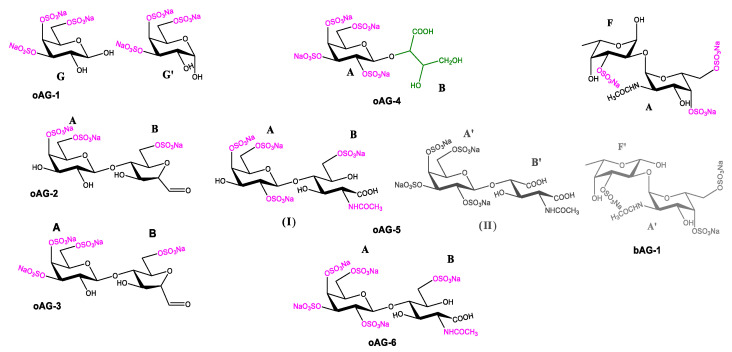
Structures of oligosaccharides oAG-1~oAG-6 and bAG-1. The letters G, G’, A, B, F and F’ in Scheme 1 represent the corresponding sugar residues in the chemical structures of oligosaccharides. These letters are also used in the 1D/2D NMR spectra. (I) and (II) are the two compounds present in oAG-5.

Structural analysis of the purified oligosaccharide oAG-1 was achieved with 1D/2D NMR spectroscopy (Figure 3 and Figure S2). The carbon number in its ^13^C NMR spectrum suggests that oAG-1 should be a monosaccharide. The resonances at 5.54 and 96.05 ppm could be assigned to the anomeric H1 and C1 of this unit. Starting from the H1 signal at 5.54 ppm in the ^1^H-^1^H COSY spectrum (Figure S2), the signals of H2, H3, H4, H5 and H6 could be sequentially attributed according to the observed spin–spin system. The H/C chemical shifts and coupling constants summarized in Table S1 suggest that it should be a sulfated galactose in α-anomer (G) according to the comparison with the data of galactose [23]. ^1^H/^13^C resonances of the β-anomer (G′) were also observed. Interestingly, the C-3, C-4 and C-6 positions should be substituted by sulfate groups based on their characteristic ^1^H/^13^C chemical shifts. Thus, oAG-1 was identified as an α/β-D-Gal_3S4S6S_ unit (Scheme 1), which should be generated during the deaminative depolymerization process, due to the possible loss of 2,5-anhydrohexose residues [24]. Additionally, the structure of oAG-1 was confirmed by the ESI-Q-TOF MS data (Figure S3).

**Figure 4:**
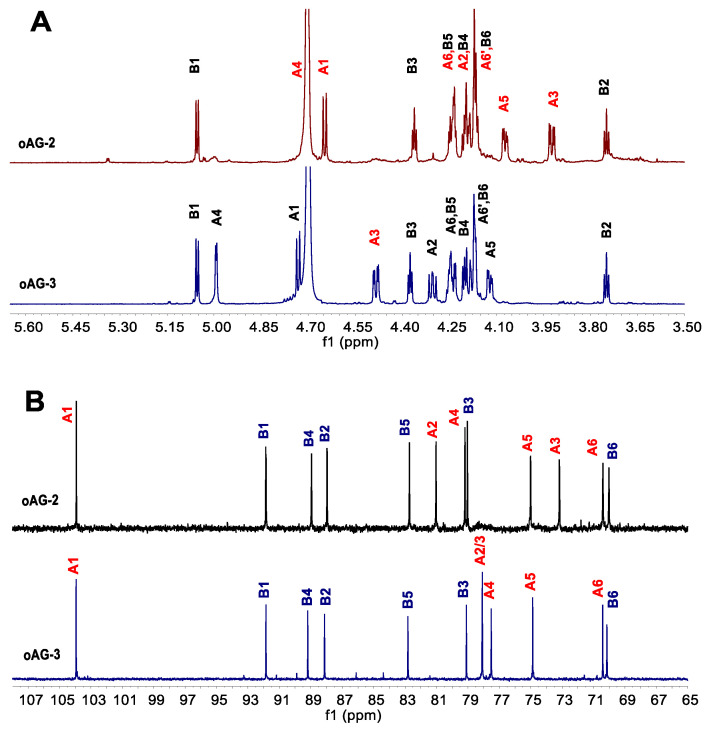
^1^H (**A**) and ^13^C (**B**) NMR spectra of oAG-2 and oAG-3.

**Figure 5:**
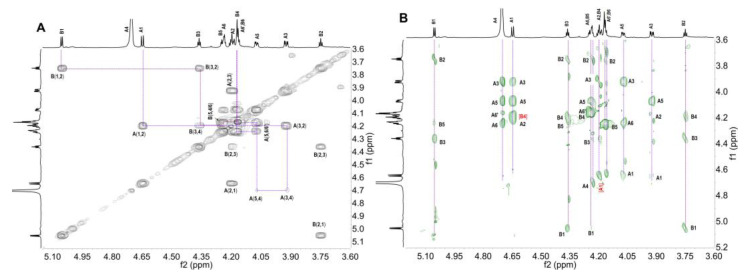

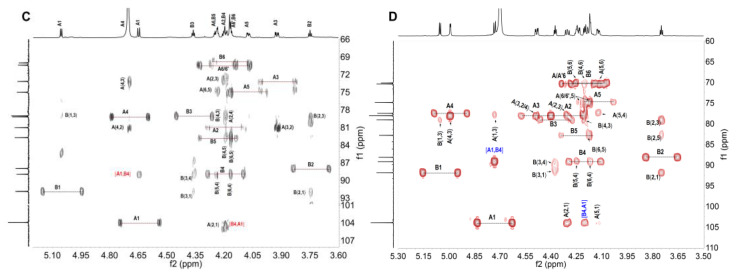
^1^H-^1^H COSY (**A**), ROESY (**B**) and ^1^H-^13^C HMBC (**C**) spectra of oAG-2 and oAG-3 (**D**).

The 1D/2D NMR spectra of oAG-2 and oAG-3 are clear and have a good resolution (Figure 4 and Figure 5), allowing their structure elucidation. The structural analysis of oAG-2 is shown as a representation. In the ^1^H-^1^H COSY spectrum (Figure 5A), two spin–spin coupling systems (A and B) could be clearly observed, suggesting that oAG-2 should be a disaccharide. Similar to the oligosaccharides produced by nitrous acid from chitosan, the new reducing end 2,5-anhydro-D-mannose (D-aMan) unit was recognized [25,26]. Obviously, the signals at 5.05 ppm (B1) in ^1^H NMR and 103.94 ppm in ^13^C NMR were from the anomeric resonances of the B unit, indicating that it should be the D-aMan residue generated by the deaminative cleavage of the D-GlcN residue. The sulfation patterns of D-aMan could be determined by the downfield shifts of the signal of C-6 (70.02 ppm). The other signals should be ascribed to the D-Gal residue (A), and the O-4, O-6 positions were bearing sulfate groups according to the downfield shifted chemical shifts of C-4 (79.20 ppm) and C-6 (70.41 ppm). The full H/C signal assignments of oAG-2 could be achieved by extensive analysis of the ^1^H-^1^H COSY, TOCSY, ROESY and ^1^H-^13^C HSQC spectra and are summarized in Table 1. Furthermore, the glycosidic bond between residues A and B could be identified from the cross peaks in ^1^H-^1^H ROESY and ^1^H-^13^C HMBC from their anomeric protons (Figure 5B,C). Specifically, the peaks of A1–B4 (4.647, 88.96 ppm) in ^1^H-^13^C HMBC, along with the large value of *J*_1,2_ (7.84) of unit A, could indicate D-Gal_4S6S_ linked to D-aMan_6S_ via the β1,4 glycosidic linkage. Thus, oAG-2 could be identified as the disaccharide with the structural sequence of β-D-Gal_4S6S_-1,4-D-aMan_6S_. Using a similar approach (Figure 4, Figure 5 and Figure S4, Table 1), complete signal assignments of oAG-3 were obtained, and oAG-3 was identified as β-D-Gal_3S4S6S_-1,4-D-aMan_6S_. The sulfation positions of the Gal residue in oAG-2 and oAG-3 were different.

### 2.3. Low-Molecular-Weight Products Produced with Free Radical Depolymerization

Free radical depolymerization induced by H_2_O_2_ and a metal catalyst is a convenient approach to obtain low-molecular-weight products with a constant composition from natural polysaccharides [27]. Then, the AG was subjected to free radical depolymerization. By controlling the reaction time, nine homogeneous components (dAG1–dAG9) with different Mws were obtained by further purification.

**Figure 6:**
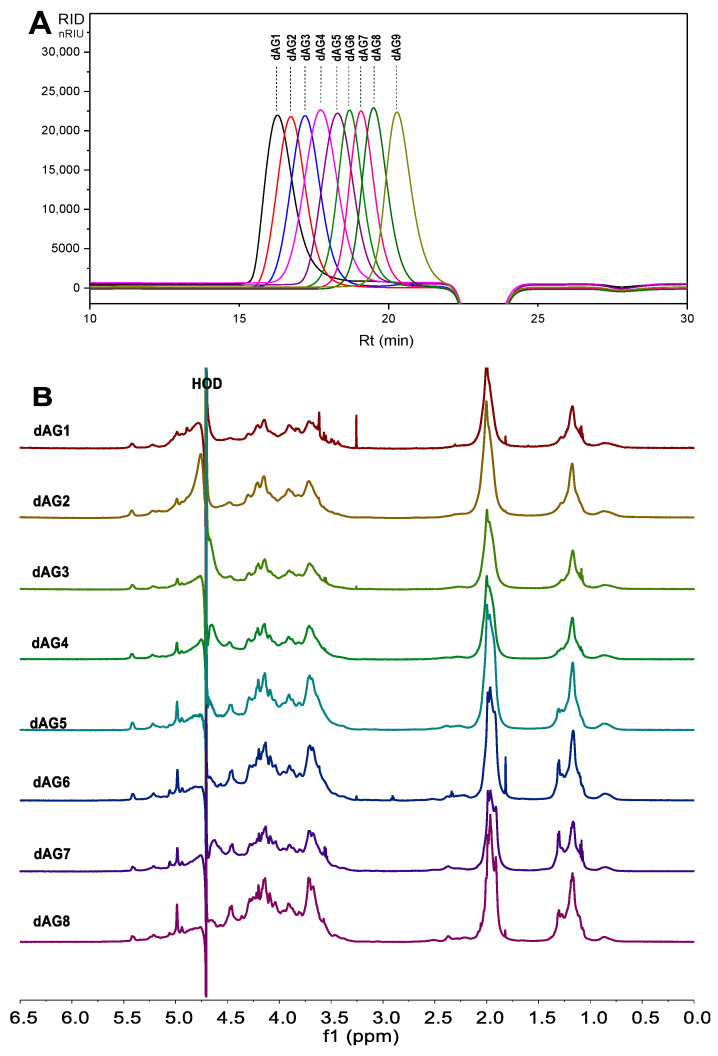
HPGPC profiles of dAG1–9 (**A**) analyzed on a Shodex OHpak SB-804 HQ (8.0 × 300 mm) column and ^1^H NMR spectra of dAG1–8 (**B**).

The molecular mass, including weight-average molecular mass (Mw), number-average molecular mass (Mn) and molecular weight distribution (Mw/Mn) of dAG1–dAG9 (Figure 6A) were determined with HPGPC [16]. The ^1^H NMR spectra of dAG1~dAG8 are shown in Figure 6B, and they remain essentially consistent with the natural AG, except that the peaks are well split.

**Figure 7:**
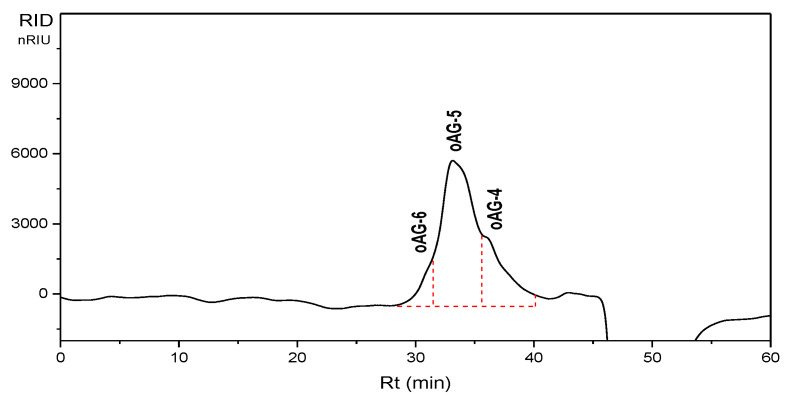
HPGPC profiles of dAG9 analyzed on a Superdex Peptide 10/300 GL column.

Particularly, since the ^1^H NMR spectrum (Figure S5) of dAG9 (Mw 1357 Da) was relatively clearer than that of dAG1–dAG8, it was further purified on a Bio-gel P6 column to afford the oligosaccharides oAG-4, oAG-5 and oAG-6 (Figure 7).

**Figure 8:**
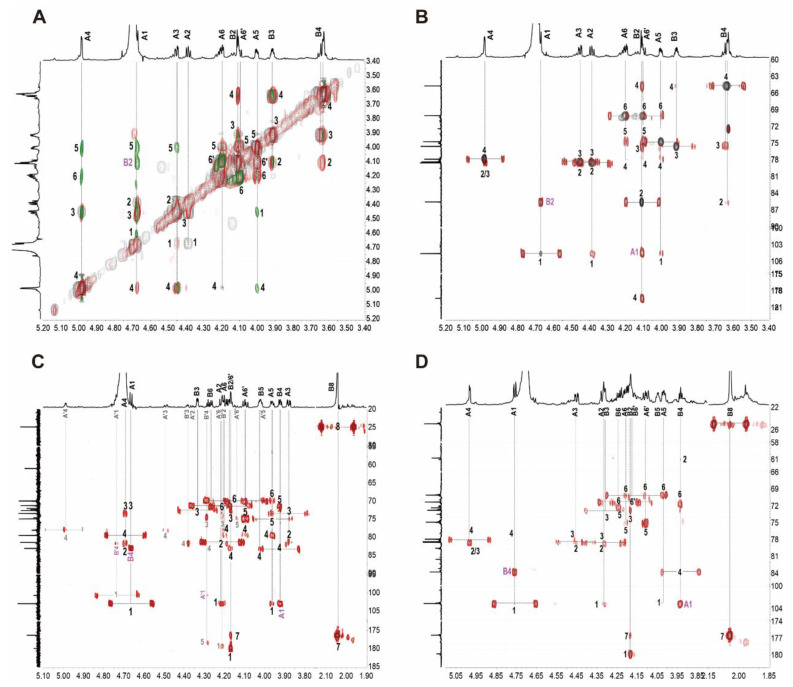
The overlapped ^1^H-^1^H COSY (black), TOCSY (red) and ROESY (green) spectra (**A**); ^1^H-^13^C HSQC/HMBC (**B**) spectra of oligosaccharide oAG-4; ^1^H-^13^C HMBC spectra of oligosaccharides oAG-5 (**C**) and oAG-6 (**D**).

The chemical structure of the oligosaccharide oAG-4 was analyzed with 1D/2D NMR spectra (Figure S6 and Figure 8). In the ^1^H NMR spectrum, the signal at 4.68 ppm was assigned to the anomeric proton of Gal (A). Moreover, the corresponding chemical shift value of C-1 was 104.51 ppm (Figure S6 and Figure 8). The downfield shifts of C2, C3, C4 and C6 indicated that Gal was highly sulfated (Table S2). According to ^1^H-^1^H COSY, TOCSY (Figure 8A) and ^1^H-^13^C HSQC (Figure 8B), the corresponding proton and carbon signals of the D-Gal_2S3S4S6S_ unit can be assigned. Additionally, three other signals were observed at 4.11 (B2), 3.91 (B3) and 3.63 (B4) ppm, and the corresponding carbons were at 85.5, 75.5 and 64.63 ppm, respectively. The connectivity of these three signals was clearly assigned in the overlapped ^1^H-^1^H COSY, TOCSY and ROESY spectra. The 64.63 ppm could be assigned to the methylene -CH_2_ carbon by comparing the ^13^C NMR and DEPT 135 spectra (Figure S6). Meanwhile, a carbonyl carbon signal at 179.30 ppm (B1) should be obtained from a carboxylate group due to the overoxidation induced by free radicals during the depolymerization process [28,29]. In the ^1^H-^13^C HMBC spectrum (Figure 8B), this carbonyl carbon showed a correlation with B2. Taken together, fragment B was identified as D-2,3,4-trihydroxybutyric acid, which should be derived from the GlcNAc_6S_ residue during free radical depolymerization. The ^1^H-^1^H ROESY and ^1^H-^13^C HMBC spectra revealed that β-D-Gal_2S3S4S6S_ was attached to position 2 of D-2,3,4-trihydroxybutyric acid (Figure 8A,B).

Similarly, the oligosaccharides oAG-5 and oAG-6 could be identified as disaccharides composed of a sulfated Gal unit and a fragment derived from the GlcNAc_6S_ unit (Figure 8C,D, Figures S7 and S8). Specifically, two disaccharides (I and II) with the structural sequence of I β-D-Gal_3S4S6S_-1,4-D-GlcUANAc_6S_ and II β-D-Gal_2S3S4S6S_-1,4-D-GlcUANAc were identified. Apart from the observed sulfated Gal residue in disaccharides oAG-2 and oAG-3, disaccharides I and II in oAG-5 contained a modified GlcNAc_6S_ at the reducing end (Scheme 1), as revealed by the cross peaks of distinct carbonyl carbons and protons, respectively. The ^1^H-^1^H ROESY and ^1^H-^13^C HMBC spectra revealed that Gal was attached to GlcUANAc_6S_ or GlcUANAc by a 1,4 linkage. The 1D/2D NMR spectra analysis indicated that oAG-6 is also a disaccharide with the structural sequence β-D-Gal_2S3S4S6S_-1,4-D-GlcUANAc_6S_. The complete signal assignments of oAG-5 and oAG-6 are demonstrated in Table S2.

The molecular formula and molecular weight of oAG-4, oAG-5 and oAG-6 were verified with ESI-Q-TOF MS (Figure S9).

### 2.4. Structure Identification of Oligosaccharide Released with Mild Acid Hydrolysis

Since fucose forms a glycosidic linkage that is more sensitive to acid than that formed by hexosamines, we attempted to defucosylate the AG using partial hydrolysis with acid. The released oligosaccharides could be used to provide additional structural information on the natural AG [7,10,30].

**Figure 9:**
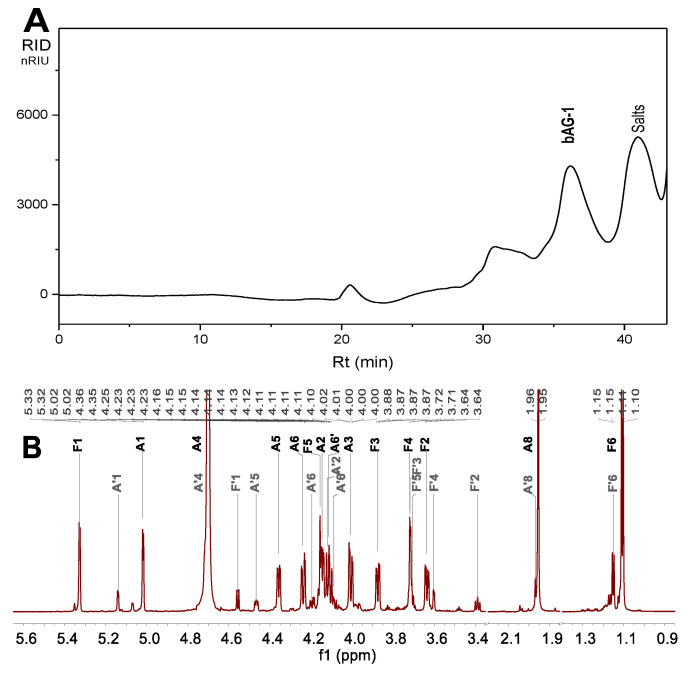

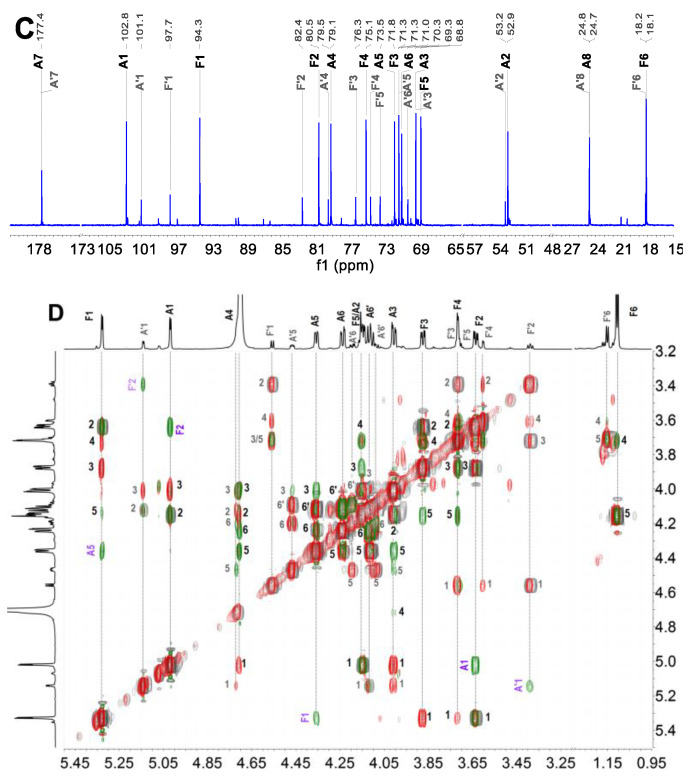
HPGPC profiles of the supernatant (**A**) from the mild acid hydrolysis of the analyzed AG on a Superdex Peptide 10/300 GL column. ^1^H (**B**).^13^C NMR (**C**). Overlapped ^1^H -^1^H COSY (gray), TOCSY (red) and ROESY (green) spectra (**D**) of the oligosaccharide bAG-1.

Under mild acid conditions, the high- and low-molecular-weight components in the hydrolysates were separated with ethanol precipitation (80%, V/V). The oligosaccharides remained in the supernatant as revealed with HPGPC analysis (Figure 9A), while the precipitate mainly contained the high-molecular-weight components (mAG). In the ^1^H NMR spectrum of mAG (Figure 1), the signals remained consistent with those of the native AG, whereas the integral ratio of the -CH_3_ at ~2.0 ppm attributed to N-acetyl hexosamine and the -CH_3_ at ~1.1 ppm of fucose units was reduced from 1:0.5 to 1:0.3, indicating that the glycosidic bonds formed by fucose were partially hydrolyzed.

The supernatant was further purified on a Bio-gel P6 column, and the major oligosaccharide component bAG-1 was obtained. The structure of bAG-1 was determined using detailed 1D/2D NMR analysis (Figure 9B–D). In the ^1^H NMR spectrum (Figure 9B) of bAG-1, the signals at 1.10–1.15 ppm could be readily assigned to the methyl protons of Fuc residues (F and F’), and the signals at ~1.95 ppm could be assigned to the methyl of the N-acetyl hexosamines present in the AG, implying that bAG-1 should be a disaccharide. In the 4.9–5.4 ppm region, three signals were observed at 5.02, 5.14 and 5.33 ppm, which could be assigned to the α-anomeric protons according to their small coupling constant (Table 2). In the overlapped ^1^H-^1^H COSY, TOCSY and ROESY spectra (Figure 9C), starting from the above α-anomeric protons, the other protons in the corresponding sugar ring could be clearly assigned. Meanwhile, a second set of signals starting from 4.556 ppm was observed, which should be assigned to H-1 of the L-Fuc residue in β-configuration. Additionally, the ^13^C NMR spectrum could be assigned through the cross peaks in the ^1^H-^13^C HSQC spectrum (Figure S10A). The ^1^H/^13^C chemical shifts are summarized in Table 2. The ^1^H/^13^C chemical shifts of the hexosamine residue identified it as α-D-GalNAc residue, according to the small coupling constant *J*_3,4_ = 2.64. The strong downfield shift observed in the F3 (3.87/71.30 ppm) of L-Fuc (F and F’), A4 (4.70/79.15 ppm) and A6 (4.23, 4.11/70.79 ppm) of D-GalNAc (A and A’) indicates the 3-sulfation of the L-Fuc unit (L-Fuc_3S_) and both the 4- and 6-sulfation of D-GalNAc (D-GalNAc_4S6S_). The correlation peaks of A1 (5.019 ppm)/F2 (3.636 ppm) and A’1 (5.141 ppm)/F’2 (3.389 ppm) in the ROESY spectrum (Figure 9D), together with the cross peaks of A1 (H1)/F2 (C-2, 80.55 ppm) and A’1 (H1)/F’2 (C-2, 82.44 ppm) observed in the HMBC spectrum (Figure S10B) demonstrate that the GalNAc residue was attached to O-2 of Fuc. The *J*_1,2_ value of A and A’ was 3.92 and 3.76, respectively, confirming that the GalNAc_4S6S_ was attached to Fuc by an α1,2 linkage.

Additionally, ESI-Q-TOF MS of bAG-1 (Figure S11) mainly showed three *m/z* peaks at 548.0367, 526.0546 and 446.0979, which could be identified as [M-Na]^−^, [M-2Na+H]^−^ and [M-SO_3_Na-Na+H]^−^, respectively, confirming a molecular formula of C_14_H_23_NNa_2_O_16_S_2_. Thus, the disaccharide (bAG-1) released from the AG with mild acid hydrolysis could be deduced to be α-D-GalNAc_4S6S_-1,2-L-α-Fuc_3S_ and α-D-GalNAc_4S6S_-1,2-β-L-Fuc_3S_. The ratio of α- and β-anomers was 4:1 based on the integral of the corresponding anomeric protons of the Fuc unit.

### 2.5. Structural Characteristic of Natural AG Using a Bottom-Up Strategy

The AG found in the sea cucumber is a sulfated polysaccharide composed of four types of monosaccharides, including GlcNAc, GalNAc, Gal and Fuc, and its sulfate content is ~37%. The ^1^H NMR of the AG showed broad and overlapping signals, which are difficult to assign. An option to avoid the complex NMR interpretations can be utilized by employing a “bottom-up” analytical strategy, where oligosaccharides with simpler structures produced with a suitable depolymerization method are analyzed separately and then assembled together through a “sum-of-the-parts” approach [31]. However, the feasibility of the bottom-up analysis of polysaccharides relies on the development of a methodology for their systematic fragmentation.

Deaminative cleavage with nitrous acid is a successful chemical method for depolymerization of heparin and heparan sulfate [21]. After hydrazinolysis of the N-acetyl hexosamine in the AG, the glycosidic linkage from this residue could be selectively cleaved in acid, and the resulting fragments were labeled with anhydrosugars at the reducing end [22]. Here, after partial deacetylation of the AG with hydrazinolysis, it was depolymerized by nitrous acid at pH 4.0. Three fragments (oAG-1, oAG-2, oAG-3) were purified, and their structures were identified. As shown in Scheme 1, oAG-1 is a highly sulfated galactose α/β-D-Gal_3S4S6S_, and oAG-2 and oAG-3 are disaccharides with the structural sequence of β-D-Gal_4S6S_-1,4-D-aMan_6S_ and β-D-Gal_3S4S6S_-1,4-D-aMan_6S_, respectively. The results indicate that sulfated Gal residues are linked to GlcNAc_6S_ through a β1,4 glycosidic bond, since the reducing end aMan_6S_ was formed from the 6-sulfated GlcNH_2_ residue during deaminative depolymerization. The observed disaccharides are consistent with the repeating disaccharides of galactose and N-acetyl glucosamine in KS [18,19], suggesting that the AG may be a type of KS found in sea cucumbers. In KS, both the galactose and N-acetyl glucosamine can be 6-O-sulfated, but in the AG, galactose is highly sulfated. Interestingly, no fragments with 2,5-anhydro-D-talose as the reducing end derived from GalNAc residues were observed, suggesting that the GalNAc should not be included in the KS-like chain, and how it is present in the AG needs further investigation.

Additionally, three derivatives of the disaccharides were separated from the free radical depolymerized products (dAG9) of the AG. The sulfation types of Gal were different from those observed in oAG-2 and oAG-3, including Gal_2S3S4S6S_ and Gal_2S4S6S_ (Scheme 1), indicating the diverse sulfation types of Gal residues in the AG. The fragments linked to the sulfated Gal should be derived from the GlcNAc by oxidation and C–C bond cleavage during free radical depolymerization, as observed in the free radical depolymerization of FCS [28].

Notably, the α-D-GalNAc_4S6S_-1,2-α/β-L-Fuc_3S_ disaccharide was purified from the released fractions of the AG with mild acid hydrolysis. The results provided the clue that the natural AG probably contained the disaccharide in the form of side chains. This conclusion could provide a rational explanation for the four types of monosaccharides composed in the AG, while no oligosaccharides with 2,5-anhydro-D-talose at the reducing end were observed in the deaminative depolymerized AG. However, this conclusion requires further investigation.

Overall, the sulfated polysaccharide (AG) found in the sea cucumber *H. fuscopunctata* is clearly composed of GlcNAc, GalNAc, Gal and Fuc, and it does not bear any uronic acid residues. According to the chemical structures of the oligosaccharides oAG-2~oAG-6, the repeating building blocks in the AG should comprise the disaccharide β-D-GalS-1,4-D-GlcNAc_6S_. Thus, the AG may be a keratan sulfate-like (KS-like) glycose*-minoglycan with more diverse modifications than those reported for KS, including the sulfation positions of the Gal residue and the possible disaccharide branches α-D-GalNAc_4S6S_-1,2-α/β-L-Fuc_3S_ linked to the KS-like chain.

### 2.6. Anticoagulant Activities

**Figure 10:**
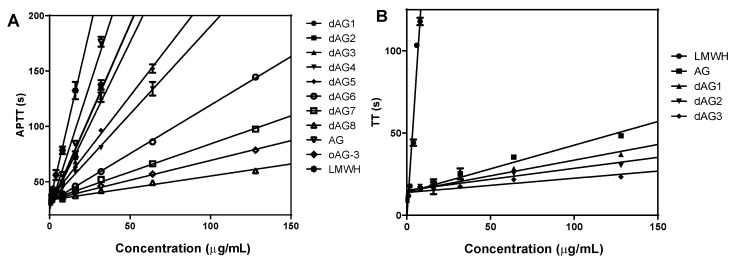
Effects of a series compounds of the AG on APTT (**A**) and TT (**B**).

It has been reported that the two sulfated fucans (SFI and SFII), a fucosylated chondroitin sulfate (FCS) and AG from *H. fuscopunctata* could prolong APTT in a dose-dependent manner [4,16]. Particularly, compared with the potent APTT prolonging activity of FCS, the AG showed a moderate APTT prolonging activity (5.84 IU/mg) [16]. Here, to elucidate the effect of Mw on the anticoagulant activity of the AG, the low-molecular-weight derivatives (dAG1~dAG9) prepared by free radical depolymerization, and three oligosaccharides (oAG-1~oAG-3) derived from the deacetylation–deaminative cleavage method were evaluated. Their effects on the APTT, prothrombin time (PT) and thrombin time (TT) of normal human plasma in vitro were analyzed and compared with those of LMWH (Table 3). The results showed that the AG, dAG1–dAG7 could prolong APTT in a dose-dependent manner (Figure 10). The natural AG exhibited significant activity in prolonging APTT (8.54 μg/mL), which is consistent with our previous results [16]. The depolymerized products dAG1-dAG5 with Mw higher than 7.5 kDa displayed similar APTT prolonging activities, while dAG6 (5.3 kDa) and dAG7 (3.7 kDa) showed weak activities, indicating that the chain length of the AG derivatives is important for maintaining their potent anticoagulant activity. Additionally, dAG8 (2.6 kDa) and dAG9 (1.4 kDa) showed negligible APTT prolonging activity. Interestingly, a highly sulfated disaccharide (oAG-3) with the structural sequence β-D-Gal_3S4S6S_-1,4-D-aMan_6S_ displayed APTT prolonging activity at a concentration of 83.99 μg/mL. Although this activity is very weak, it can be suggested that this distinct structural sequence may provide some anticoagulant activity.

Regarding the activities in prolonging TT, the AG could also prolong the TT, and the concentration of the AG required to double TT was 15.75 μg/mL, which was two times weaker than its activity in prolonging APTT. dAG1, dAG2 and dAG3 could prolong TT (Figure 10B), and they doubled the TT at 19.63, 23.85 and 44.97 μg/mL, respectively. No significant effects of dAG4~dAG9 nor oAG-1~oAG-3 were observed at concentrations as high as 128 μg/mL. Moreover, all the AG related derivatives in this study showed no influence on PT at the highest concentration of 128 μg/mL. The above results indicate that the AG could inhibit the intrinsic and common coagulation pathways, while it showed no effect on the extrinsic coagulation pathway. With the reduction in Mw, the effect on the common coagulation pathway decreased more than the effect on the intrinsic coagulation pathway. Nevertheless, further studies are needed to investigate the detailed structure-activity relationship of oligosaccharides derived from the AG.

## 3. Materials and Methods

### 3.1. Materials and Reagents

A natural AG (purity: 99.9% measured with HPGPC, average molecular weight: 39.60 kDa) was isolated from the sea cucumber *H. fuscopunctata*. Amberlite FPA98 Cl ion-exchange resin was purchased from Rohm and Haas Co. Hydrazine hydrate (containing about 64 wt% hydrazine in water) was obtained from Aladdin Reagent (Shanghai, China). Hydrazine sulfate and NaNO_2_ were purchased from Damao Chem. Ltd. (Tianjin, China). D_2_O (99.9% atom D) was from Cambridge isotope laboratories (Andover, MA, USA). Agilent 1260 HPLC system was from Agilent Technologies (Santo Clara, CA, USA). Sephadex G25/50 and Bio-gel P10/P6 were from GE Healthcare (Chicago, IL, USA) and Bio-Rad Laboratories (Hercules, CA, USA), respectively. All other reagents were commercially available and of analytical grade. The APTT, PT and TT were determined with a coagulometer (TECO MC-4000, Neufahrn, Germany) using APTT, PT and TT reagents and normal human plasma.

### 3.2. Extraction and Purification of Natural AG

The extraction of crude polysaccharide from the body wall of the sea cucumber *H. fuscopunctata* was performed as described previously [16]. After preliminary purification on a FPA98 column, the high-molecular-weight fucan sulfate (SFI) was removed and the component containing FCS, SFII and the natural AG was collected and subjected to the β-eliminative depolymerization to cleave FCS into low-molecular oligomers. The high-molecular components (mFCS, >30 kDa) containing the AG were enriched by membrane separation on a Pellicon Mini device (Millipore) with a 0.1 m^2^ membrane with MWCO of 30 kDa. For further purification of the AG, briefly, 35 g mFCS was applied to an anion-exchange FPA98 resin column and eluted with NaCl gradient solutions (1.3 M, 1.5 M, 1.8 M). The polysaccharide in the 1.8 M NaCl eluate was obtained after desalination and lyophilization, which showed a symmetrical HPGPC peak. Further monosaccharide composition was analyzed according to the PMP derivation HPLC method [16]. The sulfate content of the AG was determined with a conductimetric titration method [16]. The nature of the AG was further revealed using ^1^H NMR spectroscopy.

### 3.3. Deaminative Depolymerization of AG and Purification of Oligosaccharide

Previous data have suggested that the AG is composed of four types of monosaccharides, including GlcNAc, GalNAc, Gal and Fuc [16]. According to the deacetylation and deaminative depolymerization approach, 2 g AG and 500 mg hydrazine sulfate were dissolved in 48 mL H_2_NNH_2_·H_2_O and reacted at 90 °C for 6 h under a nitrogen atmosphere. The solution was then cooled, precipitated with ethanol (80%, *v*/*v*) and centrifuged (3500× *g* 15 min), and this step was repeated four times. The final precipitate was dissolved in 40 mL H_2_O and dialyzed against H_2_O with a dialysis bag (MWCO 500–1000 Da, Spectrum Laboratories Inc., Piscataway, NJ, USA). The retentate was lyophilized, and a partially deacetylated AG (deAG) was obtained [21,22], and the degree of deacetylation was identified with the ^1^H NMR spectrum.

The pH 4 nitrous acid reagent was prepared by adding 0.5 M H_2_SO_4_ to 5.5 M NaNO_2_. A total of 80 mL of pre-cooled nitrous acid reagent was added to the partially N-deacetylated AG solution (40 mL), and the solution was kept at 0 °C for 10 min. The reaction was terminated by neutralization with 0.5 M NaOH. The final mixture was dialyzed against water (MWCO 500–1000 Da) and then lyophilized to provide the depolymerized products with dAG ~700 mg.

dAG was dissolved in 5 mL of 0.2 M NaCl and applied to a column packed with Bio-gel P10 and eluted with 0.2 M NaCl solution. The elution profile was monitored at 210 nm. Some of the fractions were applied to HPGPC analysis equipped with a Superdex Peptide 10/300 GL column (GE Healthcare). Elution was performed with 0.2 M NaCl solution at a flow rate of 0.4 mL/min. Chromatography was recorded by a refractive index detector (RID). After repeated GPC (Bio-gel P6) separation, three oligosaccharides comprising oAG-1, oAG-2 and oAG-3 were collected, desalted by Sephadex G10, and used for 1D/2D NMR analysis.

### 3.4. Free Radical Depolymerization

To elucidate the structures and anticoagulant activity of the AG, the low-molecular-weight products were prepared by free radical depolymerization [32]. Briefly, the AG (1.8 g) and 15.12 mg of copper (II) acetate monohydrate were dissolved in 64.8 mL H_2_O. Then, 1.11 g sodium acetate and 470.0 mg NaCl were added to the solution. After the addition of 18.0 mL of 30% H_2_O_2_, the mixture was stirred at 50 °C for 12 h. Samples of 15 mL were taken at 4, 6 and 12 h, and precipitated by the addition of four volumes of ethanol (80%, *v*/*v*). Each crude depolymerized product was collected as a precipitate after centrifugation at 4000 rpm for 20 min, and repeated precipitation was performed against ethanol four times. The remaining free and associated Cu^2+^ ions were exchanged with a cation exchange resin column (001 × 732^#^, Tianjin Bohong Resin Technology Co., Ltd., Tianjin, China). The eluate was concentrated, desalted on a Sephadex G25 column (3.0 × 150 cm) and lyophilized to obtain the depolymerization products.

The abovementioned depolymerized products were further fractionated using a Sephadex G50 column (2.0 × 150 cm). The elution curve was detected with UV-Vis absorption at 210 nm. High-molecular-weight components were collected and further purified repeatedly with GPC using Sephadex G100, according to the retention time measured on an Agilent 1260 HPLC system equipped with a Shodex OH-pak SB-804 HQ column. Finally, nine homogeneous fractions dAG1–dAG9 were obtained after desalting. Their molecular weights were then determined using the HPGPC method described in Section 3.6.

In particular, dAG9 was fractionated with GPC using Bio-gel P6 column, and three homogeneous fractions oAG-4, oAG-5 and oAG-6 were obtained for further structure elucidation [28].

### 3.5. Mild Acid Hydrolysis of AG

For the presence of Fuc residues in the AG, a native AG was subjected to mild acid hydrolysis procedures, which could hydrolyze the glycosidic bonds formed by Fuc to generate fragments [7]. Here, 703 mg of the AG was treated with 140 mL H_2_SO_4_ (0.1 M) at 80 °C for 0.5 h. After cooling to room temperature, the reaction solution was neutralized with 6 M NaOH, followed by fractionation with ethanol (80%, *v*/*v*). After centrifugation, the fragments in the supernatant and the components in the precipitate were separated, and detected with HPGPC. The supernatant was desalted on a Sephadex G10 column, lyophilized and purified on Bio-gel P6 (medium, 2 × 160 cm). The collected fractions were monitored by absorbance at 210 nm and analyzed using a Superdex Peptide 10/300 GL column (GE Healthcare) on an Agilent 1260 HPLC system equipped with RID. Finally, the oligosaccharide fractions were collected and desalted with Sephadex G10 to obtain bAG-1, which was analyzed with 1D/2D NMR spectroscopy.

### 3.6. Determination of Homogeneity and Molecular Weight

The molecular mass, including weight-average molecular mass (Mw), number-average molecular mass (Mn) and molecular weight distribution (Mw/Mn) of dAG1–dAG9 were determined with HPGPC according to a previous method [16]. For molecular weight calculation, the Shodex OH-pak SB-804 HQ column (8 × 300 mm) was calibrated with standard D-series dextrans (D-0, 1, 2, 3, 4, 5, 6, 7, 8 and 2000) and a FCS fraction from *H. fuscopunctata* with known molecular weight (Mw 27,760 Da, Mn 24,380 Da) determined by size exclusion chromatography with multiple angle laser light scattering [33].

### 3.7. 1D/2D NMR and ESI-Q-TOF MS Spectroscopy

Each sample (5–10 mg) was dissolved in 0.5 mL 99.9% D_2_O and exchanged three times in 99.9% D_2_O. One-dimensional (1D) and two-dimensional (2D) C/H correlation NMR spectra (^1^H-^1^H COSY, TOCSY, ROESY, ^1^H-^13^C HSQC and HMBC) were measured at 298 K on a Bruker Advance 800 MHz spectrometer equipped with a ^13^C/^1^H dual probe in FT mode. Some ^1^H NMR spectra were recorded on a Bruker Advance 600 MHz spectrometer, which have been illustrated in corresponding figures. All the chemical shifts of ^1^H/^13^C are relative to sodium 3-trimethyl [D4] propionate (TSP-d4) [34].

ESI-Q-TOF MS analysis was performed on an Agilent 6540 UHD Accurate-Mass Q-TOF LC/MS spectrometer as previously described [35].

### 3.8. Anticoagulant Activity

The anticoagulant activities of the natural AG, the series of free radical depolymerized products dAG1–dAG9 and the oligosaccharides comprising oAG-1–oAG-3 were assessed [4,33]. The commercial APTT, PT and TT reagents and standard human plasma were used to measure APTT, PT and TT on a coagulometer (TECO MC-4000, Berlin, Germany) as previously described [36]. Low-molecular-weight heparin (enoxaparin, LMWH) was used as a positive control. A series of concentrations (640, 320, 160, 80, 40, 20 and 10 μg/mL) were prepared for each sample. The concentration of the compound required to double the clotting time was calculated. Experiments were performed in duplicate.

## 4. Conclusions

Here, a sulfated polysaccharide (AG) was purified from the sea cucumber *H. fuscopunctata* with strong anion exchange chromatography and chemical depolymerization methods. The AG was composed of four types of monosaccharides, including GlcNAc, GalNAc, Gal and Fuc. The sulfate content of the AG was ~37% as determined using a conductivity titration method. Additionally, several chemical depolymerization methods, including deaminative depolymerization, free radical depolymerization and mild acid hydrolysis, were applied to obtain oligosaccharides and then used to elucidate the structure of the AG using a bottom-up strategy. The compounds oAG-2~oAG-6 were the disaccharide derivatives of β-D-GalS-1,4-D-GlcNAc_6S_, indicating that the the repeating building blocks in the AG should comprise β-D-GalS-1,4-D-GlcNAc_6S_. Interestingly, the α-D-GalNAc_4S6S_-1,2-α/β-L-Fuc_3S_ disaccharide was identified from the mild acid hydrolysates of the AG, which should be present in the form of branches in the AG. Thus, the AG may be a keratan sulfate-like (KS-like) glycosaminoglycan with more diverse modifications than those reported for KS, including the sulfation types of the Gal residue and the possible disaccharide branches α-D-GalNAc_4S6S_-1,2-α/β-L-Fuc_3S_ linked to the KS-like chain. However, the position of branches cannot be established in this study since the oligosaccharides with a high degree of polymerization were difficult to obtain.

The anticoagulant activities of the AG, a series of low-molecular-weight homogeneous compounds comprising dAG1~dAG9 and three fragments oAG-1~oAG-3 were evaluated in vitro using control human plasma. The AG could prolong APTT in a dose-dependent manner, and AG, dAG1, dAG2 and dAG3 could prolong TT, although they have little influence on PT. The results demonstrated that the anticoagulant activity of the AG was mainly associated with the inhibition of the intrinsic and common coagulation pathway. The anticoagulant structure–activity relationship of the AG and a series of depolymerization products were further investigated. Mw is an important factor in maintaining the APTT prolonging activity of AGs. This study provides insight into the structural diversity of polysaccharides in sea cucumbers.

## Figures and Tables

**Table 1:** ^1^H/^13^C chemical shifts of oAG-2 and oAG-3.

oAG-2					oAG-3				
A, β-D-Gal_4S6S_-1,					A, β-D-Gal_3S4S6S_-1,				
H1	4.647	*J*_(1,2)_ = 7.84	C1	103.94	H1	4.732	*J*_(1,2)_ = 7.68	C1	103.96
H2	4.197	*J*_(2,3)_ = 10.00	C2	81.03	H2	4.303	*J*_(2,3)_ = 10.0	C2	78.09
H3	3.923	*J*_(3,4)_ = 3.28	C3	73.18	H3	4.484	*J*_(3,4)_ = 3.12	C3	78.10
H4	4.993	*J*_(4,5)_ = --	C4	79.20	H4	4.993	*J*_(4,5)_ = --	C4	77.52
H5	4.073	*J*_(5,6)_ = 3.36	C5	75.01	H5	4.121	*J*_(5,6)_ = 3.20	C5	74.88
H6	4.242	*J*_(6,6′)_ = 10.80	C6	70.41	H6	4.239	*J*_(6,6′)_ = 10.56	C6	70.42
H6′	4.172	*J*_(5,6′)_ = 8.64			H6′	4.182	*J*_(5,6′)_ = 8.80		
B, -4-D-aMan_6S_					B, -4-D-aMan_6S_				
H1	5.054	*J*_(1,2)_ = 5.84	C1	91.88	H1	5.054	*J*_(1,2)_ = 5.92	C1	91.87
H2	3.748	*J*_(2,3)_ = 5.52	C2	87.99	H2	3.749	*J*_(2,3)_ = 5.12	C2	88.14
H3	4.361	*J*_(3,4)_ = 4.48	C3	79.04	H3	4.375	*J*_(3,4)_ = 4.32	C3	79.10
H4	4.696	*J*_(4,5)_ = 5.20	C4	88.96	H4	4.201	*J*_(4,5)_ = 5.76	C4	89.21
H5	4.234	*J*_(5,6′)_ = 4.32	C5	82.73	H5	4.249	*J*_(5,6′)_ = 5.36	C5	82.83
H6	4.168		C6	70.02	H6	4.169	*J*_(5,6)_ = 3.36	C6	70.15

**Table 2:** ^1^H/^13^C chemical shifts of the branches bAG-1.

bAG-1					bAG-1				
A α-D-GalNAc_4S6S_-1-					A’ α-D-GalNAc_4S6S_-1-				
H1	5.019	*J*_(1,2)_ = 3.92	C1	102.77	H1	5.141	*J*_(1,2)_ = 3.76	C1	101.05
H2	4.147	*J*_(2,3)_ = 11.20	C2	52.28	H2	4.123	*J*_(2,3)_ = 11.20	C2	53.17
H3	4.006	*J*_(3,4)_ = 2.64	C3	69.34	H3	4.006	*J*_(3,4)_ = 2.64	C3	69.34
H4	4.706	/	C4	79.15	H4	4.718		C4	79.45
H5	4.358	*J*_(5,6)_ = 2.80 *J*_(5,6′)_ = 8.88	C5	71.80	H5	4.466	*J*_(5,6)_ = 4.56 *J*_(5,6′)_ = 7.92	C5	71.28
H6	4.238	/	C6	70.79	H6	4.195	*J*_(6,6′)_ = 10.88	C6	70.26
H6′	4.112	*J*_(6,6′)_ = 11.44			H6′	4.089	/		
			C7	177.39				C7	177.39
H8	1.954	*/*	C8	24.71	H8	1.969	*/*	C8	24.75
F -2-α-L-Fuc_3S_					F’ 2-β-L-Fuc_3S_				
H1	5.327	*J*_(1,2)_ = 3.92	C1	94.29	H1	4.556	*J*_(1,2)_ = 7.76	C1	97.72
H2	3.636	*J*_(2,3)_ = 10.32	C2	80.55	H2	3.389	*J*_(2,3)_ = 10.00	C2	82.44
H3	3.875	*J*_(3,4)_ = 3.20	C3	71.30	H3	3.716	*J*_(3,4)_ = 3.44	C3	76.28
H4	3.717	/	C4	75.08	H4	3.602	/	C4	74.56
H5	4.153	*J*_(5,6)_ = 6.56	C5	68.75	H5	4.705	*J*_(5,6)_ = 6.48	C5	73.46
H6	1.106	/	C6	18.14	H6	1.151		C6	18.23

**Table 3:** Average molecular weight of a series of depolymerized products of the AG and their anticoagulant activities.

Sample	Mw ^a^ (kDa)	Mn ^a^ (kDa)	PD	2 × APTT ^b^ (μg/mL)	2 × PT (μg/mL)	2 × TT ^b^ (μg/mL)
LMWH	4.4	/	/	5.20 ± 0.06	>128	1.38 ± 0.07
AG	39.1	31.2	1.25	8.54 ± 0.08	>128	15.75 ± 3.82
dAG1	35.9	30.0	1.20	11.37 ± 0.24	>128	19.63 ± 1.04
dAG2	26.7	22.1	1.21	11.11 ± 0.29	>128	23.85 ± 0.17
dAG3	18.6	15.6	1.19	12.27 ± 0.48	>128	44.97 ± 0.81
dAG4	12.3	10.0	1.23	19.97 ± 1.05	>128	>128
dAG5	7.5	6.3	1.19	16.10 ± 0.46	>128	>128
dAG6	5.3	4.5	1.18	36.45 ± 0.14	>128	>128
dAG7	3.7	3.1	1.19	59.42 ± 1.91	>128	>128
dAG8	2.6	2.2	1.18	>128	>128	>128
dAG9	1.4	1.2	1.17	>128	>128	>128
oAG-3	0.73	0.73	1	83.99 ± 1.33	>128	>128
oAG-2	0.63	0.63	1	>128	>128	>128
oAG-1	0.48	0.48	1	>128	>128	>128

^a^ Mw and Mn were determined with HPGPC. ^b^ Concentrations required for doubling APTT or TT.

## Data Availability

All data are contained in this article and the Supplementary Materials.

## Supplementary Materials

The following supporting information can be downloaded at: https://www.mdpi.com/article/10.3390/md21120632/s1, Figure S1. HPGPC profiles of mFCS and AG; Figure S2. ^1^H-^1^H COSY (A), TOCSY (B) and ROESY (C) spectra of oAG-1; Figure S3. ESI-Q-TOF MS of oAG-1; Figure S4. ^1^H-^1^H COSY (A), ^1^H-^1^H TOCSY (B), ^1^H-^1^H ROESY (C) and ^1^H-^13^C HSQC (D) spectra of oAG-3; Figure S5. ^1^H NMR spectrum of dAG-9; Figure S6. ^1^H/^13^C NMR spectra of the oligosaccharide oAG-4; Figure S7. Overlapped ^1^H-^1^H COSY (gray), TOCSY (red), ROESY (green) spectra (A), ^1^H-^13^C HSQC (B), ^1^H -^13^C HSQC (black)/HSQC-TOCSY (green) spectra of oAG-5 (C); Figure S8. Overlapped ^1^H-^1^H COSY (gray), TOCSY (red) and ROESY (green) spectra of oAG-6 (A); ^1^H-^13^C HSQC spectrum of oAG-6 (B); HSQC-TOCSY spectrum of oAG-6 (C); Figure S9. ESI-Q-TOF MS spectra of oAG-4 (A), oAG-5 (B) and oAG-6 (C); Figure S10. ^1^H-^13^C HSQC (A) and HMBC (B) spectra of bAG-1; Figure S11. ESI-Q-TOF MS of bAG-1; Table S1. ^1^H and ^13^C chemical shifts of oAG-1; Table S2. ^1^H/^13^C chemical shifts of the oligosaccharides oAG-4, oAG-5 and oAG-6.

## References

[1] Li H., Yuan Q., Lv K., Ma H., Gao C., Liu Y., Zhang S., Zhao L. (2021). Low-molecular-weight fucosylated glycosaminoglycan and its oligosaccharides from sea cucumber as novel anticoagulants: A review. Carbohydr. Polym..

[2] Xu H., Zhou Q., Liu B., Chen F., Wang M. (2022). Holothurian fucosylated chondroitin sulfates and their potential benefits for human health: Structures and biological activities. Carbohydr. Polym..

[3] Pomin V.H. (2015). Marine non-glycosaminoglycan sulfated glycans as potential pharmaceuticals. Pharmaceuticals.

[4] Sun H., Gao N., Ren L., Liu S., Lin L., Zheng W., Zhou L., Yin R., Zhao J. (2020). The components and activities analysis of a novel anticoagulant candidate dHG-5. Eur. J. Med. Chem..

[5] Zhao L., Wu M., Xiao C., Yang L., Zhou L., Gao N., Li Z., Chen J., Chen J., Liu J. (2015). Discovery of an intrinsic tenase complex inhibitor: Pure nonasaccharide from fucosylated glycosaminoglycan. Proc. Natl. Acad. Sci. USA.

[6] Cai Y., Yang W., Li X., Zhou L., Wang Z., Lin L., Chen D., Zhao L., Li Z., Liu S. (2019). Precise structures and anti-intrinsic tenase complex activity of three fucosylated glycosaminoglycans and their fragments. Carbohydr. Polym..

[7] Yin R., Zhou L., Gao N., Lin L., Sun H., Chen D., Cai Y., Zuo Z., Hu K., Huang S. (2021). Unveiling the disaccharide-branched glycosaminoglycan and anticoagulant potential of its derivatives. Biomacromolecules.

[8] Shang F., Mou R., Zhang Z., Gao N., Lin L., Li Z., Wu M., Zhao J. (2018). Structural analysis and anticoagulant activities of three highly regular fucan sulfates as novel intrinsic factor Xase inhibitors. Carbohydr. Polym..

[9] Pereira M.S., Mulloy B., Mourão P.A.S. (1999). Structure and anticoagulant activity of sulfated fucans: Comparison between the regular, repetitive, and linear fucans from echinoderms with the more heterogeneous and branched polymers from brown algae. J. Biol. Chem..

[10] Kim S.B., Farrag M., Mishra S.K., Misra S.K., Sharp J.S., Doerksen R.J., Pomin V.H. (2023). Selective 2-desulfation of tetrasaccharide-repeating sulfated fucans during oligosaccharide production by mild acid hydrolysis. Carbohydr. Polym..

[11] Yu L., Xu X., Xue C., Chang Y., Ge L., Wang Y., Zhang C., Liu G., He C. (2013). Enzymatic preparation and structural determination of oligosaccharides derived from sea cucumber (*Acaudina molpadioides*) fucoidan. Food Chem..

[12] Chang Y., Hu Y., Yu L., McClements D.J., Xu X., Liu G., Xue C. (2016). Primary structure and chain conformation of fucoidan extracted from sea cucumber *Holothuria tubulosa*. Carbohydr. Polym..

[13] Yu L., Xue C., Chang Y., Xu X., Ge L., Liu G., Wang Y. (2014). Structure elucidation of fucoidan composed of a novel tetrafucose repeating unit from sea cucumber *Thelenota ananas*. Food Chem..

[14] Luo L., Wu M., Xu L., Lian W., Xiang J., Lu F., Gao N., Xiao C., Wang S., Zhao J. (2013). Comparison of physicochemical characteristics and anticoagulant activities of polysaccharides from three sea cucumbers. Mar. Drugs.

[15] Zheng W., Zhou L., Lin L., Cai Y., Sun H., Zhao L., Gao N., Yin R., Zhao J. (2019). Physicochemical characteristics and anticoagulant activities of the polysaccharides from sea cucumber *Pattalus mollis*. Mar. Drugs.

[16] Gao N., Chen R., Mou R., Xiang J., Zhou K., Li Z., Zhao J. (2020). Purification, structural characterization and anticoagulant activities of four sulfated polysaccharides from sea cucumber *Holothuria fuscopunctata*. Int. J. Biol. Macromol..

[17] Gao L., Xu C., Tao X., Zuo Z., Ning Z., Wang L., Gao N., Zhao J. (2022). Structure elucidation of fucan sulfate from sea cucumber *Holothuria fuscopunctata* through a bottom-up strategy and the antioxidant activity analysis. Int. J. Mol. Sci..

[18] Caterson B., Melrose J. (2018). Keratan sulfate, a complex glycosaminoglycan with unique functional capability. Glycobiology.

[19] Pomin V.H. (2015). Keratan sulfate: An up-to-date review. Int. J. Biol. Macromol..

[20] Ustyuzhanina N.E., Bilan M.I., Nifantiev N.E., Usov A.I. (2019). Structural analysis of holothurian fucosylated chondroitin sulfates: Degradation versus non-destructive approach. Carbohydr. Res..

[21] Bienkowski M.J., Conrad H.E. (1985). Structural characterization of the oligosaccharides formed by depolymerization of heparin with nitrous acid. J. Biol. Chem..

[22] Shaklee P.N., Conrad H.E. (1984). Hydrazinolysis of heparin and other glycosaminoglycans. Biochem. J..

[23] Agrawal P.K. (1992). NMR Spectroscopy in the structural elucidation of oligosaccharides and glycosides. Phytochemistry.

[24] Bezrodnykh E.A., Blagodatskikh I.V., Kulikov S.N., Zelenikhin P.V., Yamskov I.A., Tikhonov V.E. (2018). Consequences of chitosan decomposition by nitrous acid: Approach to non-branched oligochitosan oxime. Carbohydr. Polym..

[25] Allison C.L., Lutzke A., Reynolds M.M. (2019). Examining the effect of common nitrosating agents on chitosan using a glucosamine oligosaccharide model system. Carbohydr. Polym..

[26] Tømmeraas K., Vårum K.M., Christensen B.E., Smidsrød O. (2001). Preparation and characterisation of oligosaccharides produced by nitrous acid depolymerisation of chitosans. Carbohydr. Res..

[27] Amicucci M.J., Nandita E., Galermo A.G., Castillo J.J., Chen S., Park D., Smilowitz J.T., German J.B., Mills D.A., Lebrilla C.B. (2020). A nonenzymatic method for cleaving polysaccharides to yield oligosaccharides for structural analysis. Nat. Commun..

[28] Chen R., Yin R., Li S., Pan Y., Mao H., Cai Y., Lin L., Wang W., Zhang T., Zhou L. (2021). Structural characterization of oligosaccharides from free radical depolymerized fucosylated glycosaminoglycan and suggested mechanism of depolymerization. Carbohydr. Polym..

[29] Panagos C., Thomson D., Bavington C.D., Uhrín D. (2012). Structural characterisation of oligosaccharides obtained by Fenton-type radical depolymerisation of dermatan sulfate. Carbohydr. Polym..

[30] Mourao P.A., Pereira M.S., Pavao M.S., Mulloy B., Tollefsen D.M., Mowinckel M.C., Abildgaard U. (1996). Structure and anticoagulant activity of a fucosylated chondroitin sulfate from echinoderm. Sulfated fucose branches on the polysaccharide account for its high anticoagulant action. J. Biol. Chem..

[31] Cegelski L. (2015). Bottom-up and top-down solid-state NMR approaches for bacterial biofilm matrix composition. J. Magn. Reson..

[32] Wu M., Xu S., Zhao J., Kang H., Ding H. (2010). Preparation and characterization of molecular weight fractions of glycosaminoglycan from sea cucumber *Thelenata ananas* using free radical depolymerization. Carbohydr. Res..

[33] Zhou L., Gao N., Sun H., Xiao C., Yang L., Lin L., Yin R., Li Z., Zhang H., Ji X. (2020). Effects of native fucosylated glycosaminoglycan, its depolymerized derivatives on intrinsic factor Xase, coagulation, thrombosis, and hemorrhagic risk. Thromb. Haemost..

[34] Mao H., Cai Y., Li S., Sun H., Lin L., Pan Y., Yang W., He Z., Chen R., Zhou L. (2020). A new fucosylated glycosaminoglycan containing disaccharide branches from *Acaudina molpadioides*: Unusual structure and anti-intrinsic tenase activity. Carbohydr. Polym..

[35] Li S., Zhong W., Pan Y., Lin L., Cai Y., Mao H., Zhang T., Li S., Chen R., Zhou L. (2021). Structural characterization and anticoagulant analysis of the novel branched fucosylated glycosaminoglycan from sea cucumber *Holothuria nobilis*. Carbohydr. Polym..

[36] Wu M., Wen D., Gao N., Xiao C., Yang L., Xu L., Lian W., Peng W., Jiang J., Zhao J. (2015). Anticoagulant and antithrombotic evaluation of native fucosylated chondroitin sulfates and their derivatives as selective inhibitors of intrinsic factor Xase. Eur. J. Med. Chem..

