# Supplementary material for: Structural Characterization and Anticoagulant Activities of a Keratan Sulfate-like Polysaccharide from the Sea Cucumber Holothuria fuscopunctata
Source: Mar Drugs. 2023 Dec 8;21(12):632. doi: 10.3390/md21120632 (PMC10744359; doi:10.3390/md21120632)
Supplement: Supplementary file 1 [file marinedrugs-21-00632-s001.zip › marinedrugs-2735723-supplementary.pdf]

Structural Characterization and Anticoagulant Activities of a Keratan Sulfate-like Polysaccharide from the Sea Cucumber *Holothuria fuscopunctata*

Ru Chen [1,2,3], Weili Wang [1,3], Ronghua Yin [4], Ying Pan [4], Chen Xu [4], Na Gao [4*], Xiaodong Luo [1,5*], Jinhua Zhao [1,4*]

1. State Key Laboratory of Phytochemistry and Plant Resources in West China, Kunming Institute of Botany, Chinese Academy of Sciences, Kunming 650201, China

2. Yunnan Institute of Traditional Chinese Medicine and Materia Medica, Kunming 650223, China

3. University of Chinese Academy of Sciences, Beijing 100049, China

4. School of Pharmaceutical Sciences, South-Central Minzu University, Wuhan 430074, China

5. Yunnan Characteristic Plant Extraction Laboratory, Key Laboratory of Medicinal Chemistry for Natural Resource, Ministry of Education and Yunnan Province, School of Chemical Science and Technology, Yunnan University, Kunming 650091, China

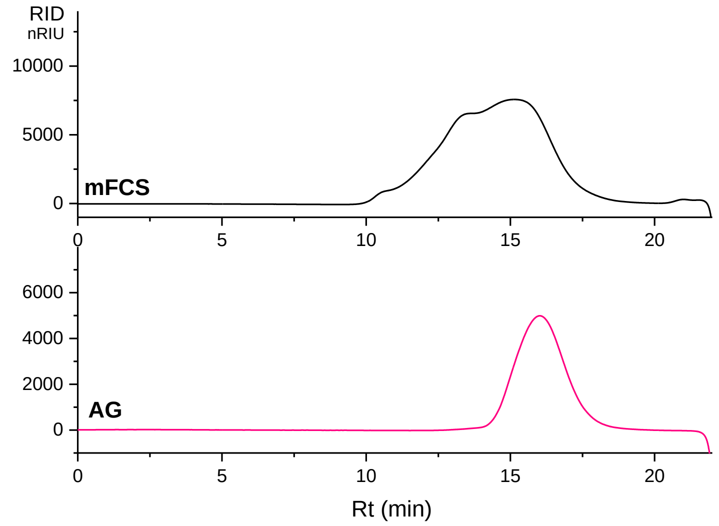

Figure S1. HPGPC profiles of mFCS and AG

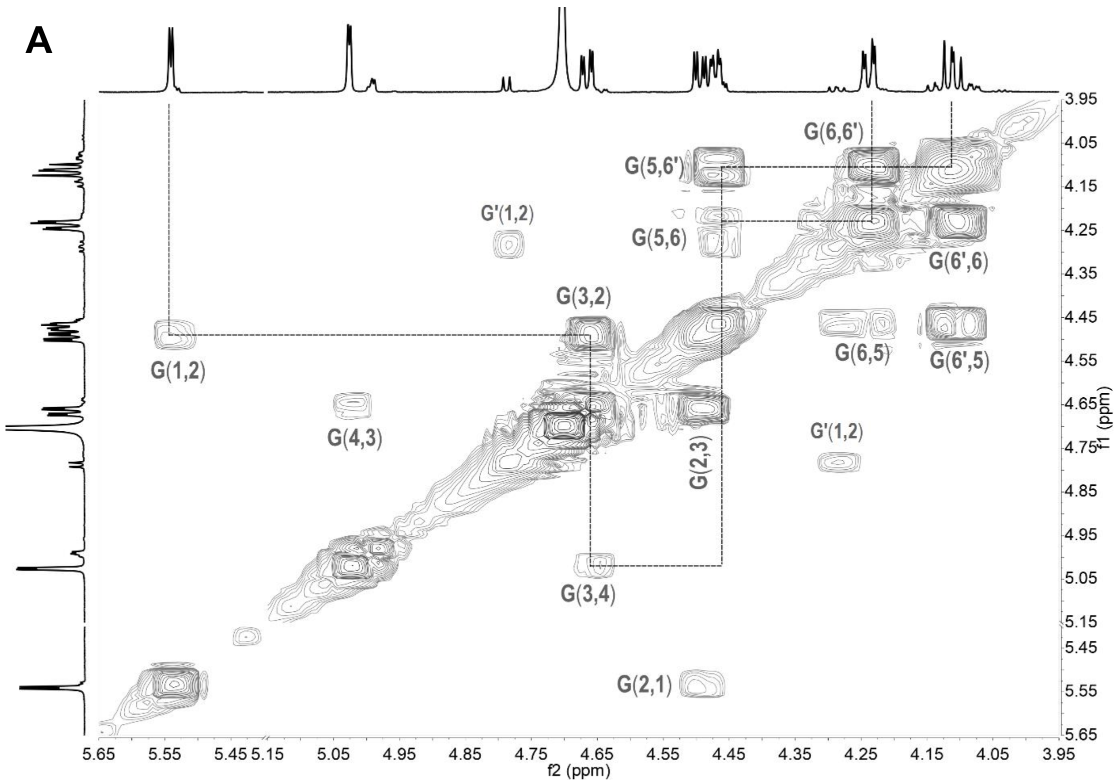

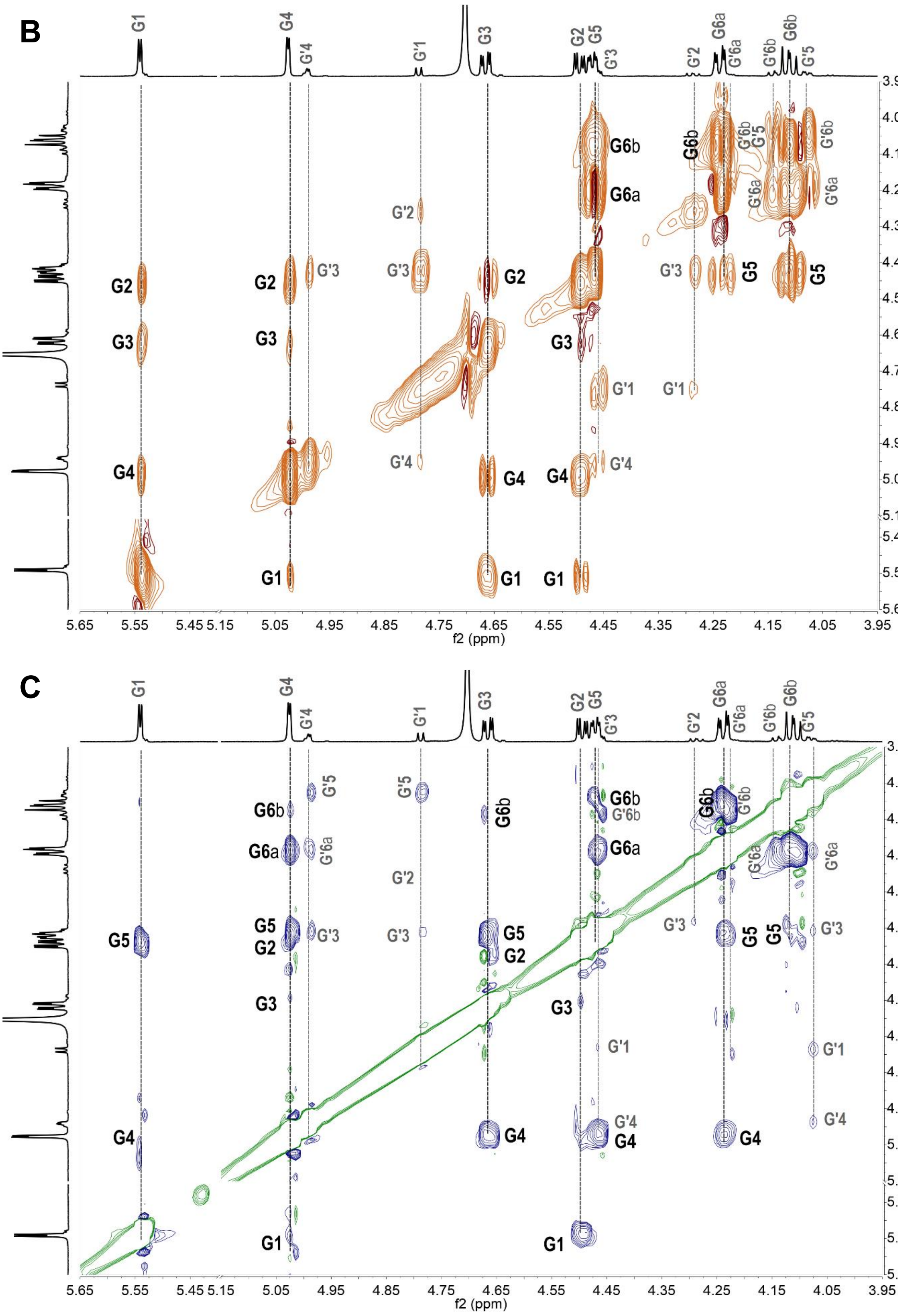

Figure S2. ^{1}H-^{1}H COSY (A), TOCSY (B) and ROESY (C) spectra of oAG-1

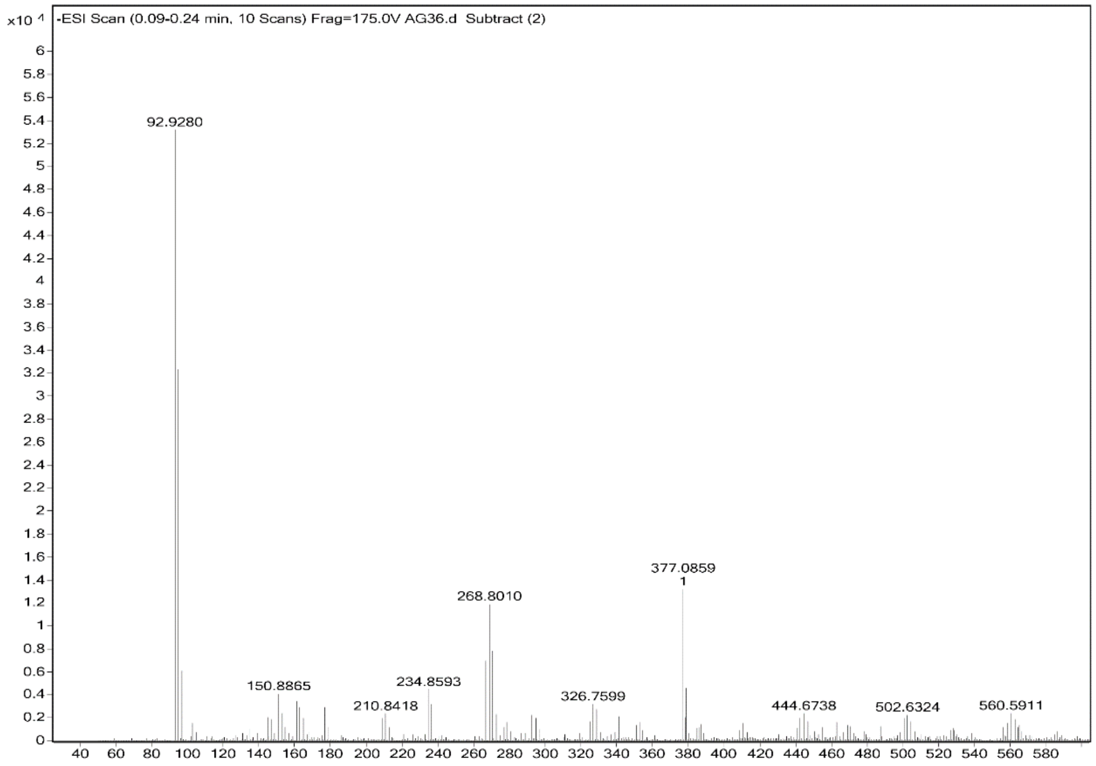

Figure S3. ESI-Q-TOF MS of oAG-1

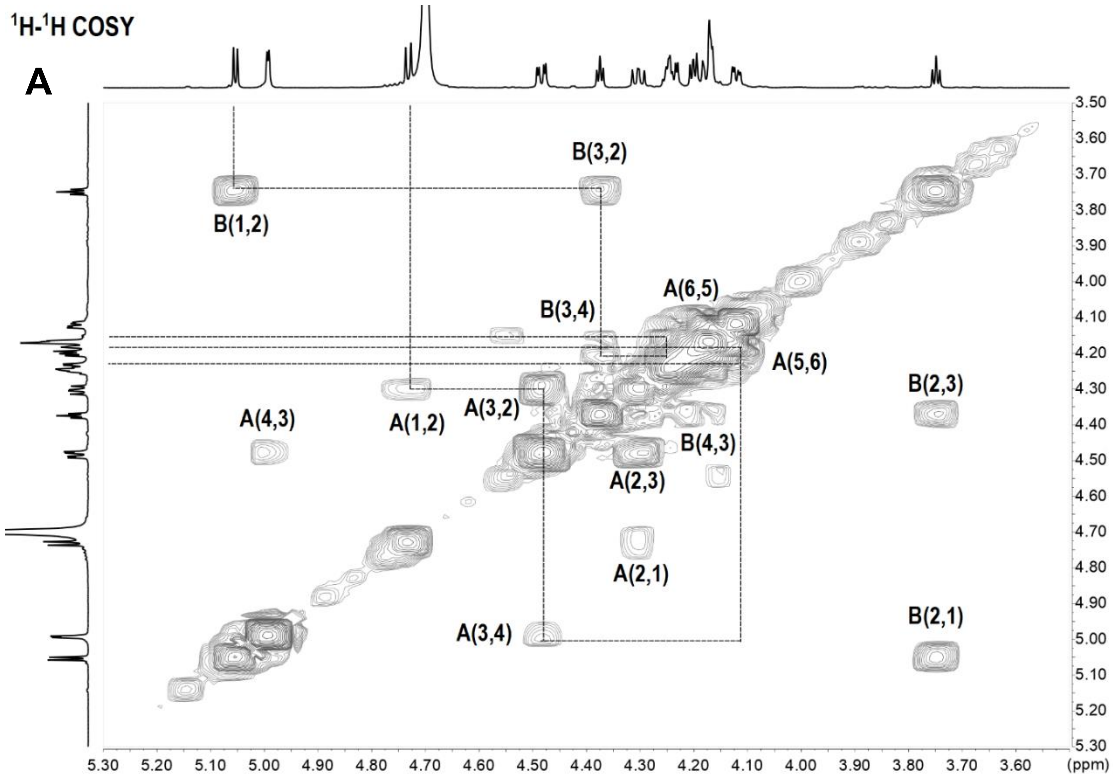

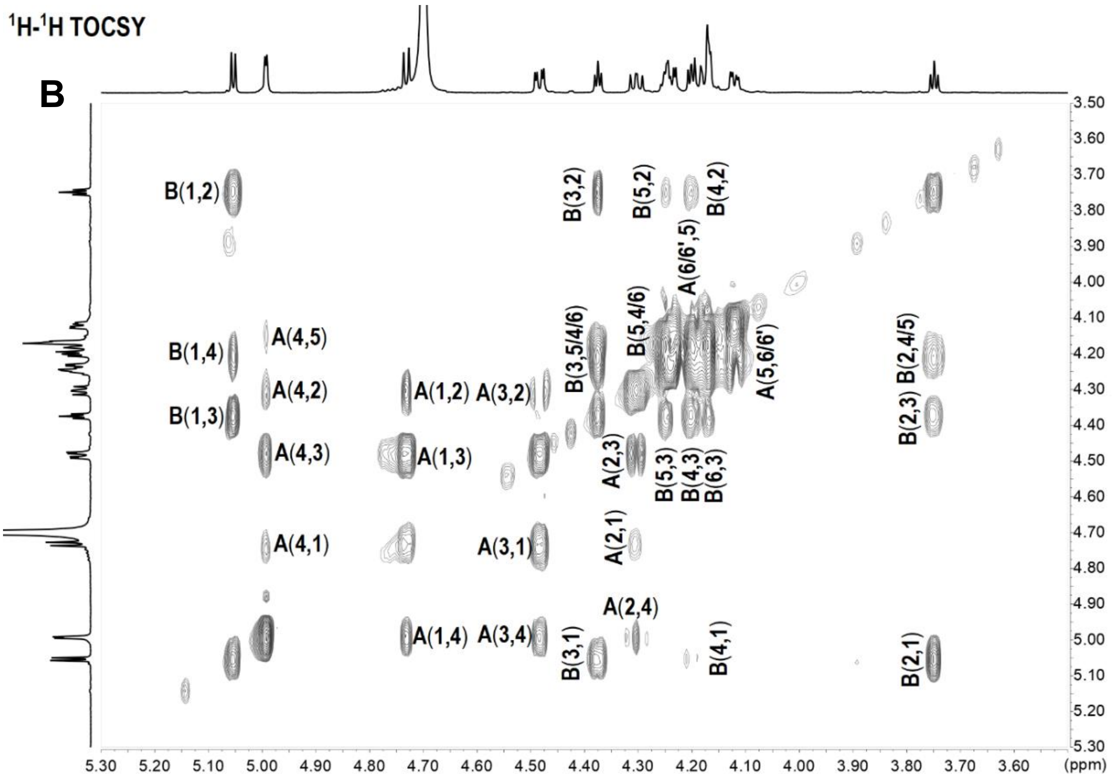

^{1}H-^{1}H TOCSY
B
B(1,2)
B(3,2)
B(5,2)
B(4,2)
A(6/6',5)
B(5,4/6)
B(3,5/4/6)
A(5,6/6')
B(2,4/5)
B(1,4)
A(4,5)
A(4,2)
B(1,3)
A(1,2)
A(3,2)
B(2,3)
A(4,3)
A(1,3)
A(2,3)
B(5,3)
B(4,3)
B(6,3)
A(4,1)
A(3,1)
A(2,1)
A(2,4)
A(1,4)
A(3,4)
B(3,1)
B(4,1)
B(2,1)
(ppm)

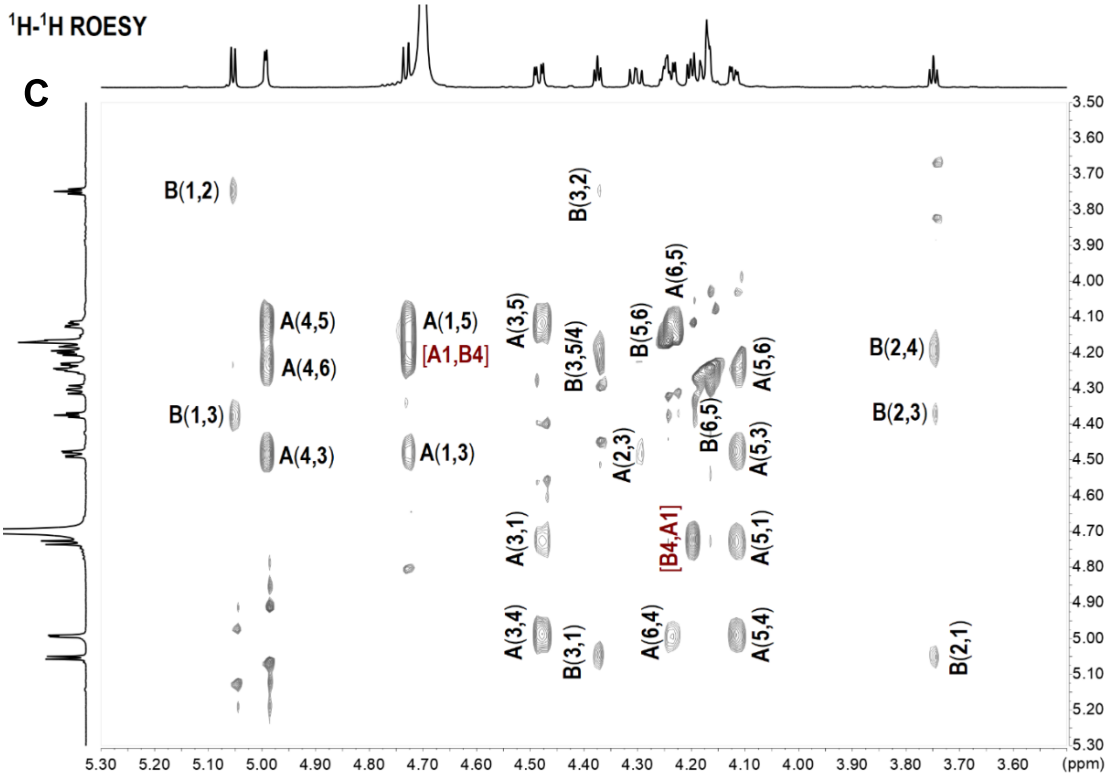

^{1}H-^{1}H ROESY
C
B(1,2)
B(3,2)
A(6,5)
A(4,5)
A(1,5)
A(3,5)
B(5,6)
[A1,B4]
B(3,5/4)
A(5,6)
B(2,4)
A(4,6)
B(1,3)
B(2,3)
B(6,5)
A(4,3)
A(1,3)
A(2,3)
A(5,3)
A(3,1)
[B4,A1]
A(5,1)
A(3,4)
A(6,4)
A(5,4)
B(3,1)
B(2,1)
(ppm)

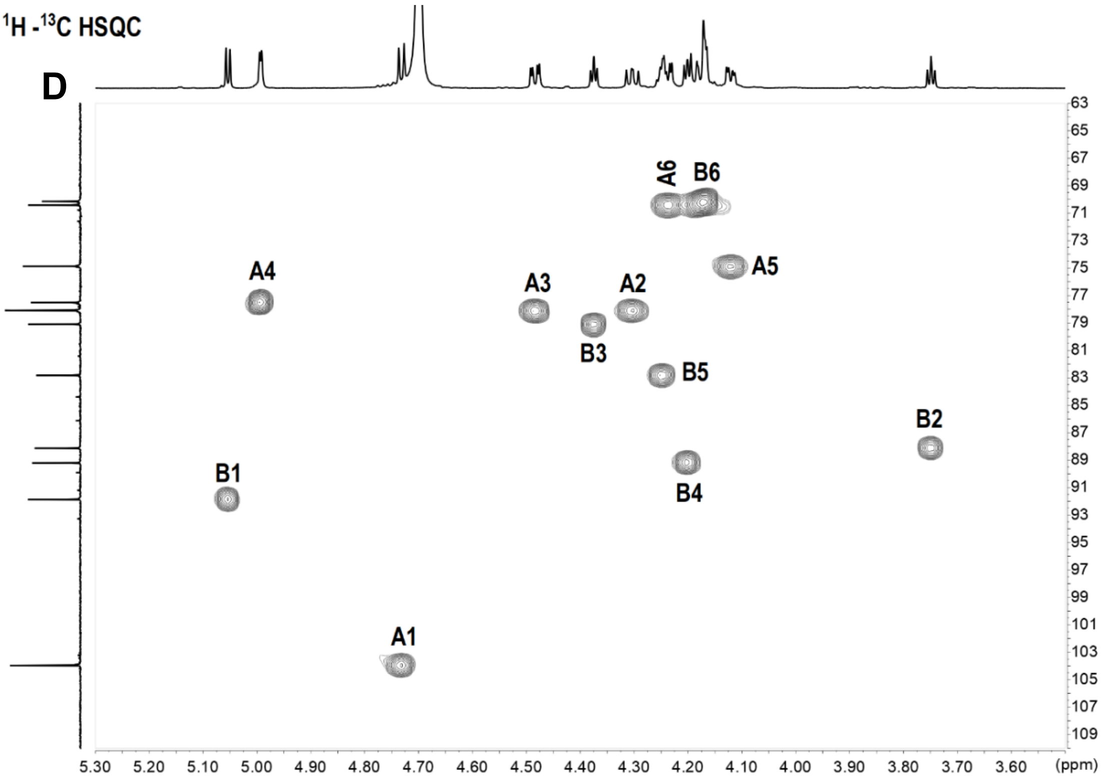

Figure S4. ^{1}H-^{1}H COSY (A), ^{1}H-^{1}H TOCSY (B), ^{1}H-^{1}H ROESY (C) and ^{1}H-^{13}C HSQC (D) spectra of oAG-3

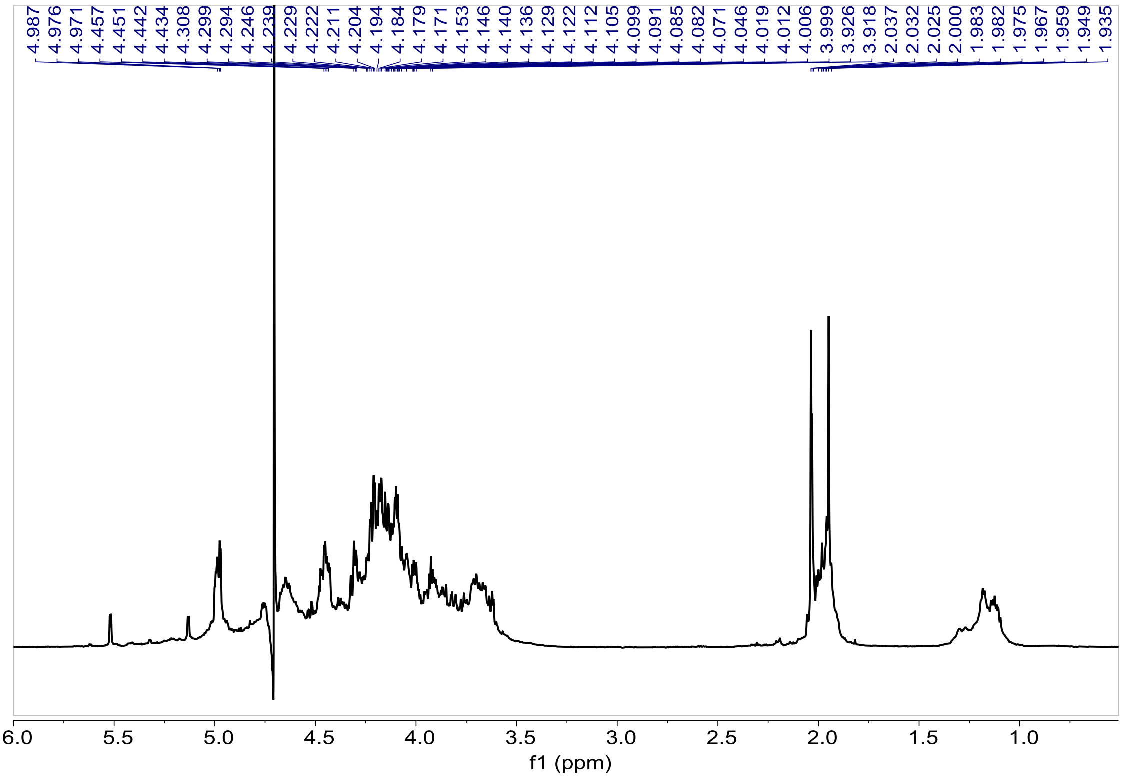

Figure S5. ^{1}H NMR spectrum of dAG9

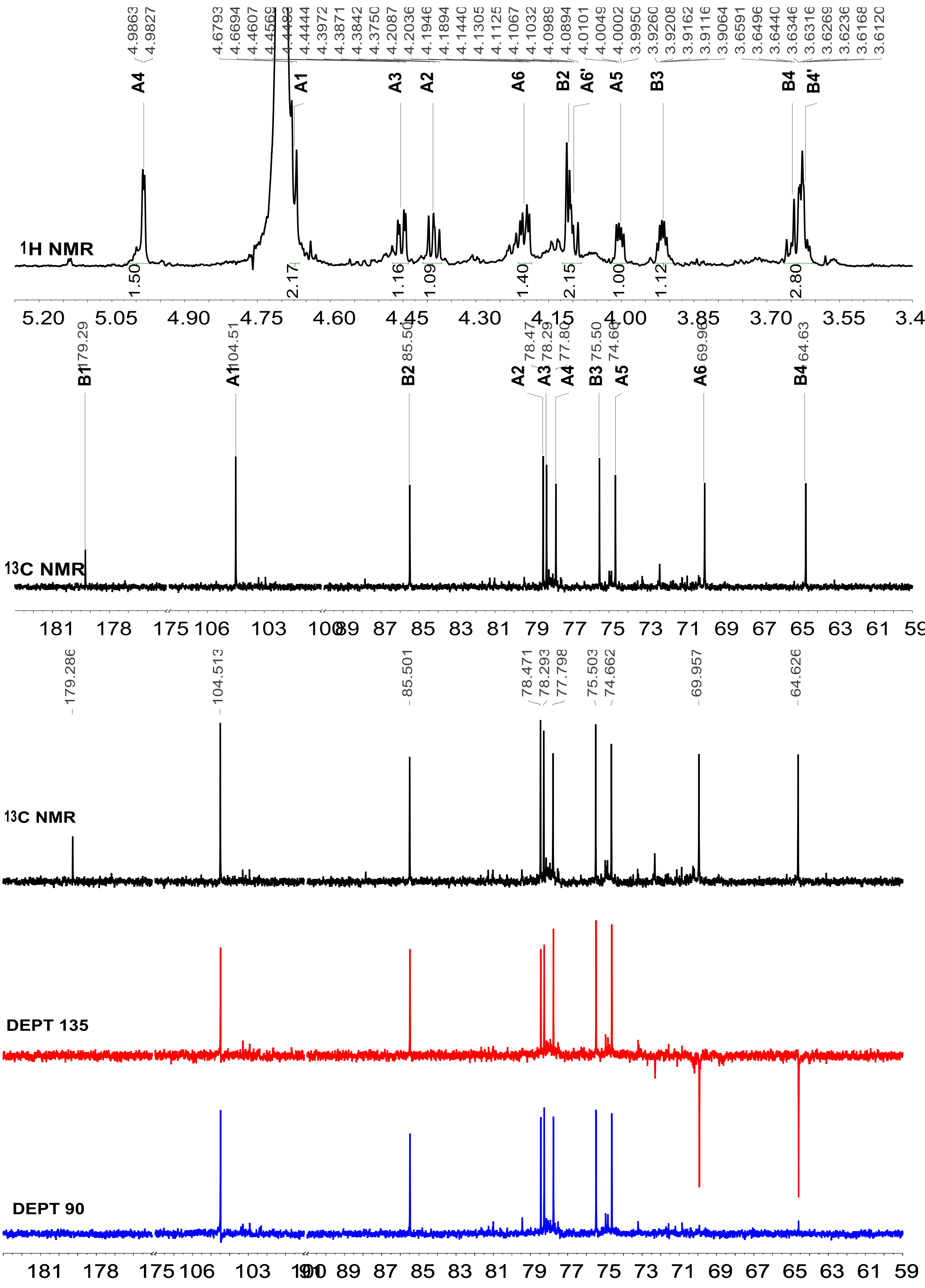

Figure S6. ^{1}H / ^{13}C NMR spectra of the oligosaccharide oAG-4

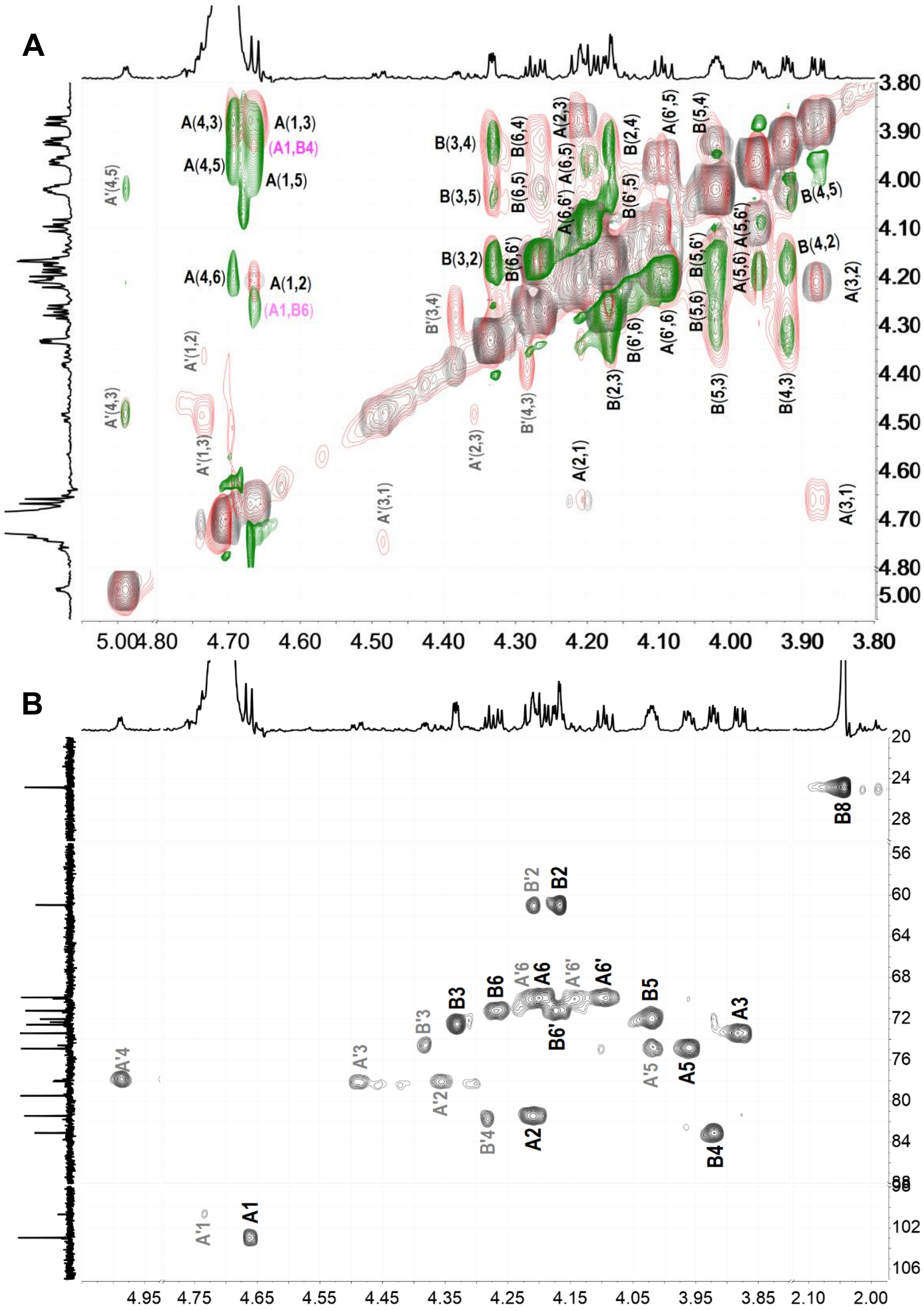

A
A(4,3)
A(1,3)
(A1,B4)
A(4,5)
A(1,5)
A'(4,5)
A(4,6)
A(1,2)
(A1,B6)
A'(1,2)
A'(4,3)
A'(1,3)
A'(3,1)
A'(2,3)
B'(3,4)
B'(4,3)
B(3,4)
B(3,5)
B(3,2)
B(6,4)
B(6,5)
B(6,6')
A(2,3)
A(6,5)
A(6,6')
B(2,4)
B(6',5)
B(6',6)
B(2,3)
A(6',5)
A(6',6)
B(5,4)
B(5,6')
B(5,6)
B(5,3)
A(5,6')
A(5,6)
B(4,5)
B(4,2)
B(4,3)
A(3,2)
A(2,1)
A(3,1)
3.80
3.90
4.00
4.10
4.20
4.30
4.40
4.50
4.60
4.70
4.80
5.00
5.004.80
4.70
4.60
4.50
4.40
4.30
4.20
4.10
4.00
3.90
3.80
B
B8
B'2
B2
A'6
A6
A'6'
A6'
B6
B3
B'3
B6'
B5
A3
A'5
A5
A'3
A'4
A'2
B'4
A2
B4
A'1
A1
20
24
28
56
60
64
68
72
76
80
84
98
102
106
4.95
4.75
4.65
4.55
4.45
4.35
4.25
4.15
4.05
3.95
3.85
2.10
2.00

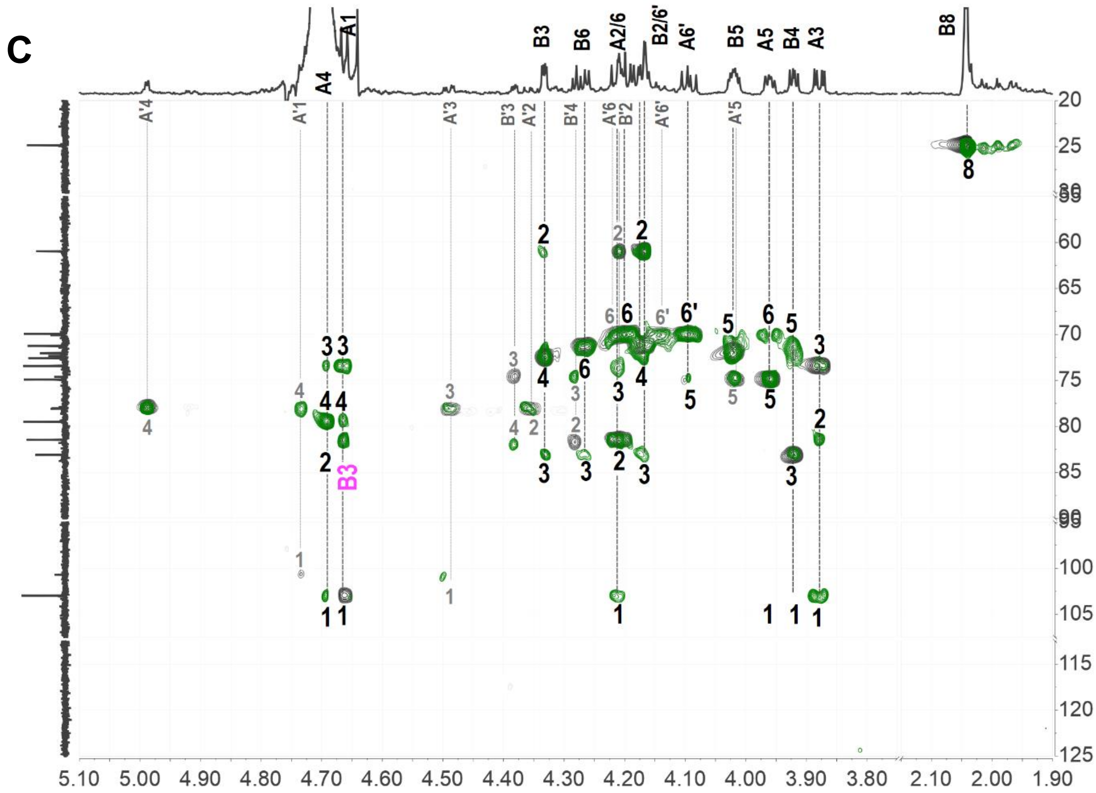

Figure S7. Overlapped ^{1}H-^{1}H COSY (gray), TOCSY (red), ROESY (green) spectra (A), ^{1}H-^{13}C HSQC (B), ^{1}H -^{13}C HSQC (black)/HSQC-TOCSY (green) spectra of oAG-5 (C)

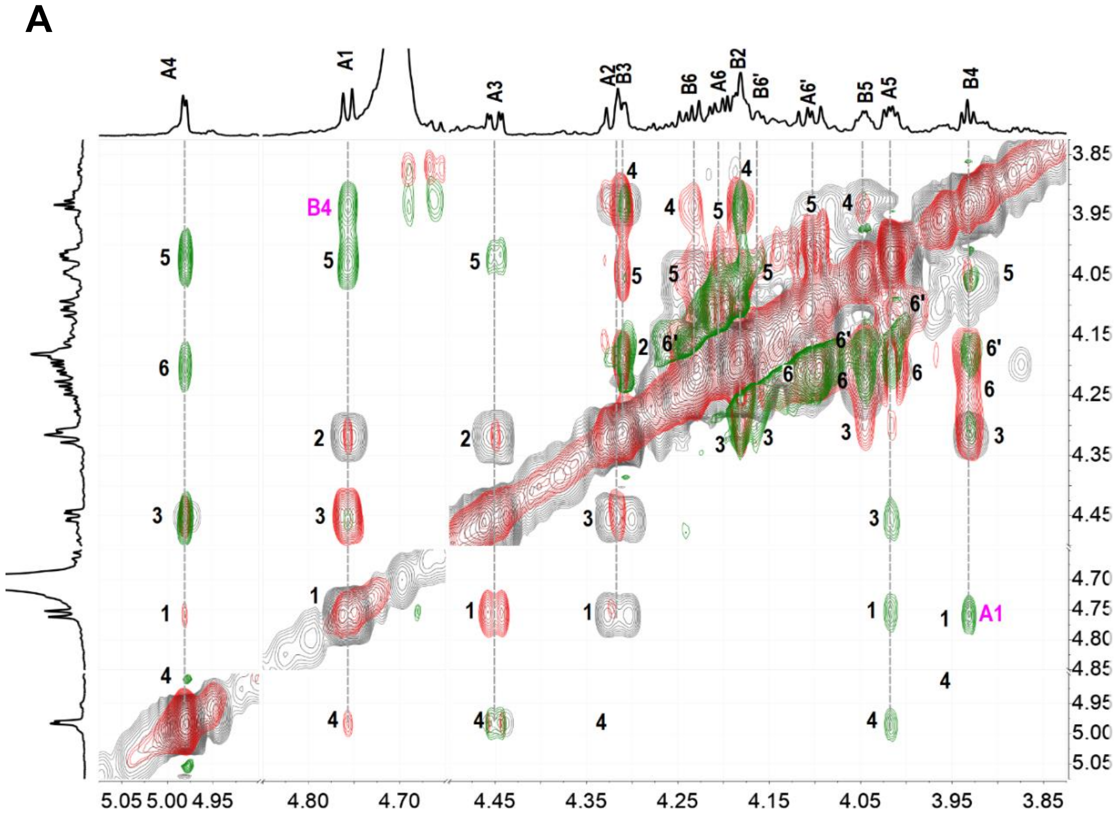

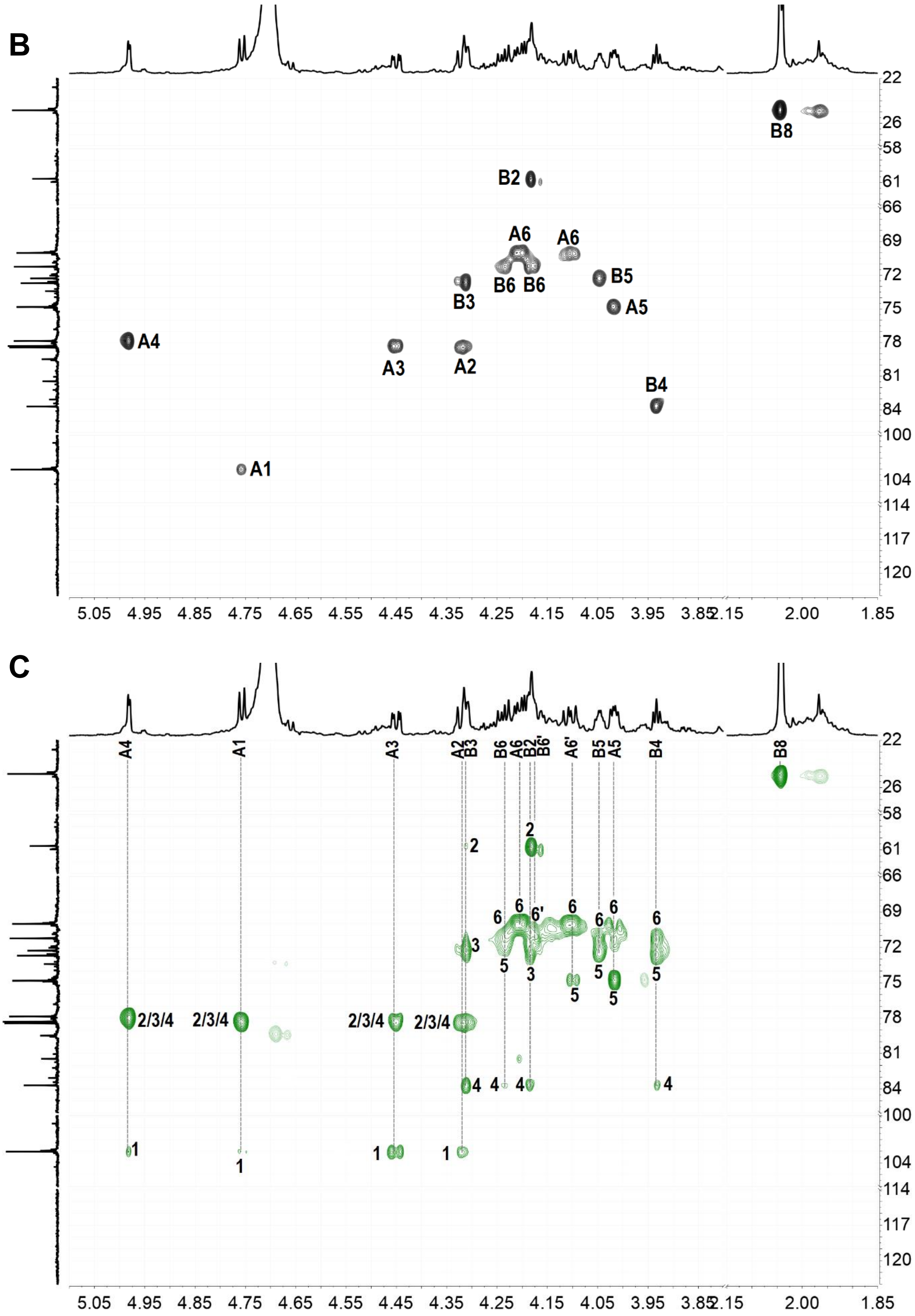

Figure S8. Overlapped ^{1}H-^{1}H COSY (gray), TOCSY (red), ROESY (green) spectra of oAG-6 (A), ^{1}H-^{13}C HSQC spectrum of oAG-6 (B) and HSQC-TOCSY spectrum of oAG-6 (C)

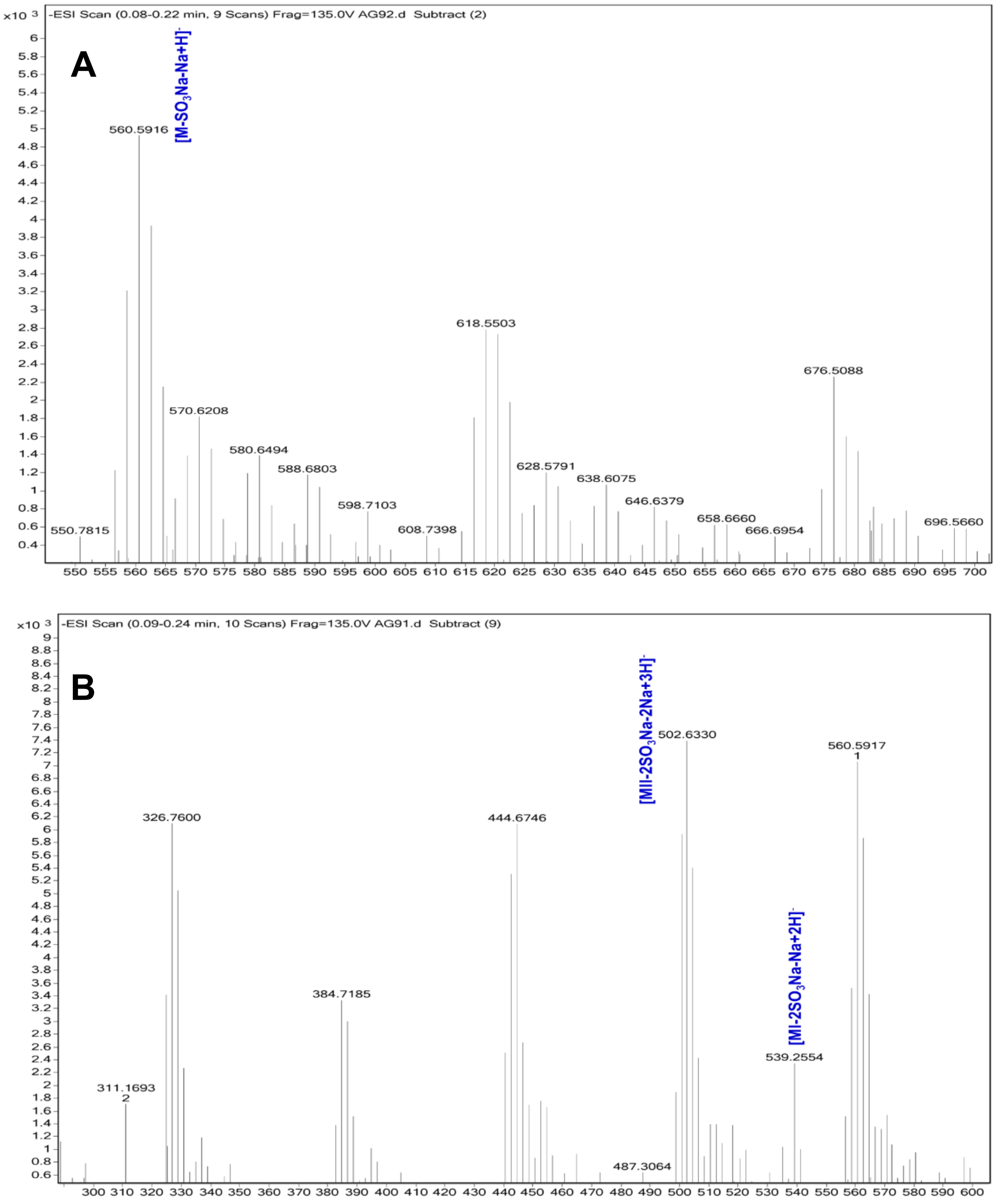
-ESI Scan (0.08-0.22 min, 9 Scans) Frag=135.0V AG92.d Subtract (2)
A
[M-SO3Na-Na+H]-
560.5916
570.6208
580.6494
588.6803
598.7103
608.7398
618.5503
628.5791
638.6075
646.6379
658.6660
666.6954
676.5088
696.5660
550.7815
-ESI Scan (0.09-0.24 min, 10 Scans) Frag=135.0V AG91.d Subtract (9)
B
[MII-2SO3Na-2Na+3H]-
502.6330
560.5917
326.7600
444.6746
384.7185
[MI-2SO3Na-Na+2H]-
539.2554
311.1693
487.3064

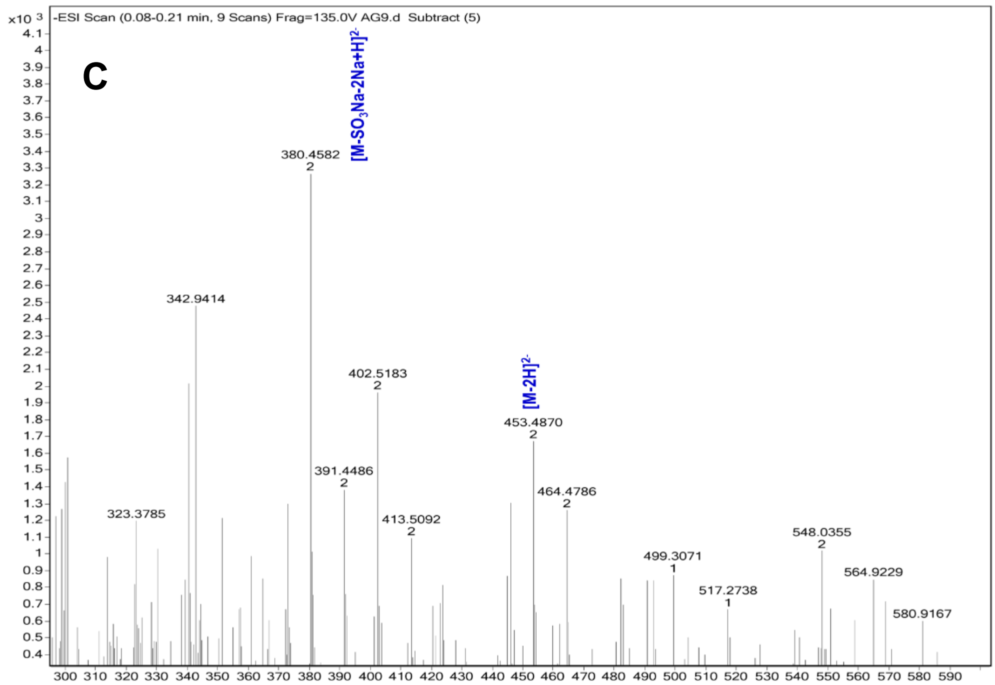

Figure S9. ESI-Q-TOF MS spectra of oAG-4 (A), oAG-5 (B) and oAG-6 (C)

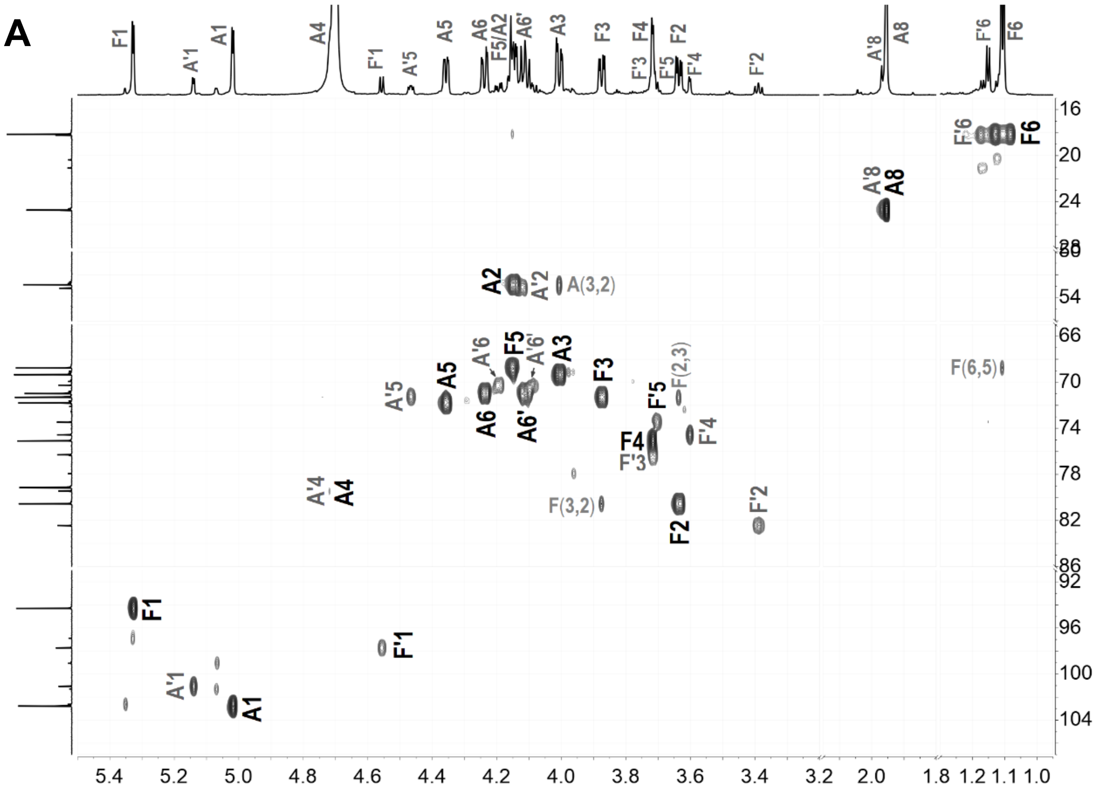

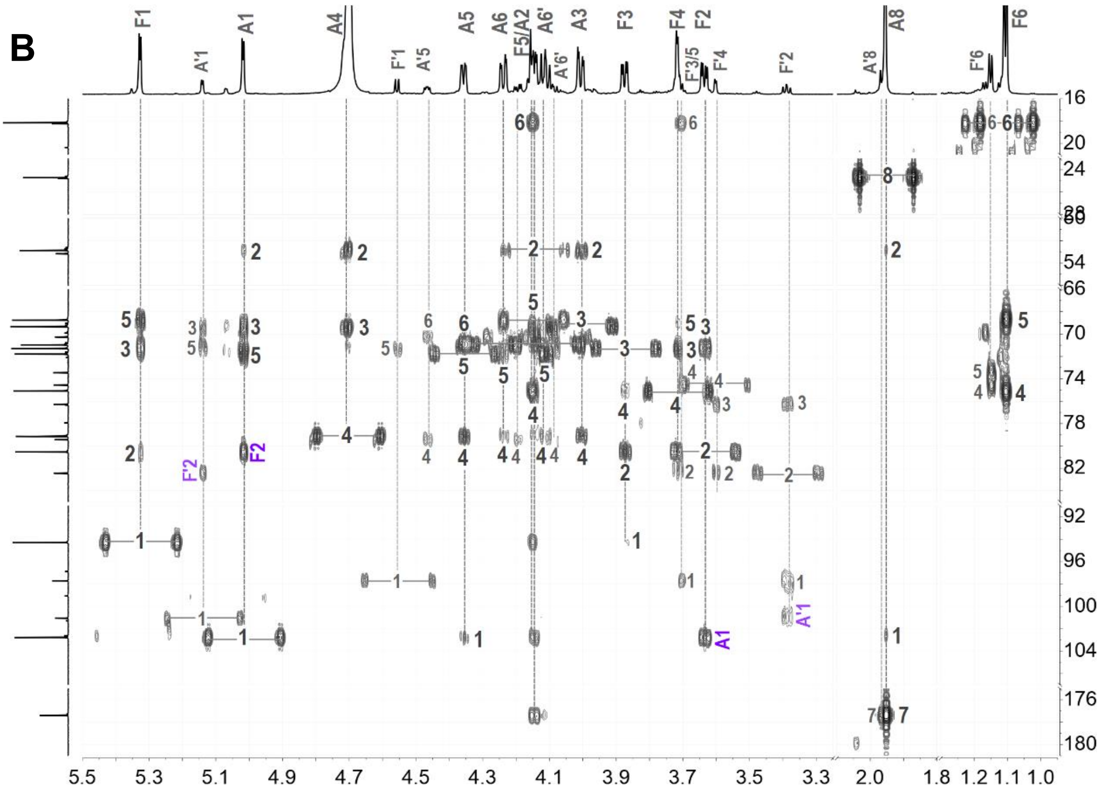

Figure S10. ^{1}H-^{13}C HSQC (A) and HMBC (B) spectra of bAG-1

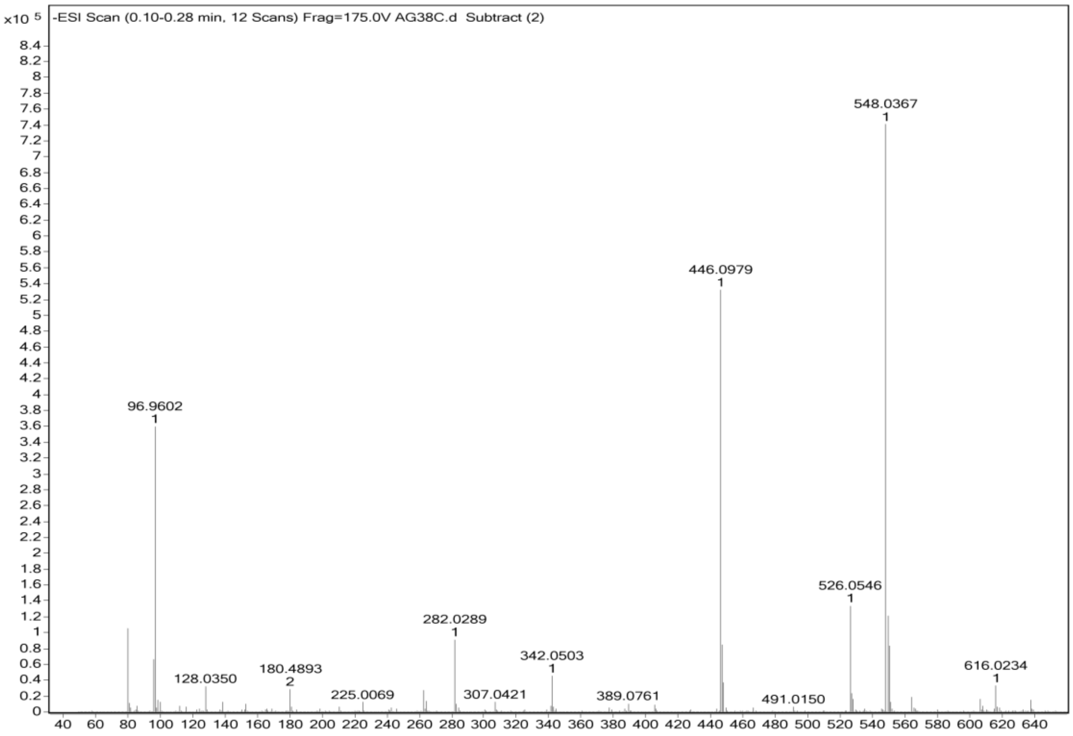

Figure S11. ESI-Q-TOF MS of bAG-1

Table S1 ^{1}H and ^{13}C chemical shifts of oAG-1

^{1}H	δ (ppm)	Coupling	COSY	TOCSY	ROESY	^{13}C	δ (ppm)	HSQC	HMBC
**G, α-D-Gal$_{3S4S6S}$**									
H1	5.541	$J_{(1,2)}$= 3.68	H2	H2,3,4	H2,4,5	C1	96.05	H1	H1
H2	4.494	$J_{(2,3)}$= 10.40	H1,3	H1,3,4	H1,3,4	C2	78.14	H2	H2,3,4
H3	4.666	$J_{(3,4)}$= 3.04	H2,4	H1,2,4	H2,4,5,6$_{b}$	C3	77.53	H3	H1,2,3,4,5
H4	5.026	$J_{(4,5)}$ = --	H3	H1,2,3	H1,2,3,5,6$_{a/b}$	C4	81.35	H4	H4
H5	4.471	$J_{(5,6)}$ = 3.20	H6$_{a/b}$	H6$_{a/b}$	H1,3,4,6$_{a/b}$	C5	73.42	H5	H1,5,6$_{a/b}$
H6a	4.238	$J_{(6,6')}$ = 11.28	H5,6$_{b}$	H5,6$_{b}$	H4,5,6$_{b}$	C6	73.34	H6	H6$_{a/b}$
H6b	4.111	$J_{(5,6')}$ = 8.96	H5,6$_{a}$	H5,6$_{a}$	H5,6$_{a}$			H6′	
**G′, β-D-Gal$_{3S4S6S}$**									
H1	4.788	$J_{(1,2)}$= 7.80	H2	H2,3,4	H3,5	C1	100.54	H1	/
H2	4.286	$J_{(2,3)}$= 10.00	H1,3	H1,3	H3	C2	82.02	H2	/
H3	4.463	$J_{(3,4)}$= 3.28	H2,4	H1,2,4	H1,4,6b	C3	80.96	H3	/
H4	4.990	$J_{(4,5)}$ = --	H3	H1,3	H3,5,6$_{a}$	C4	80.52	H4	/
H5	4.079	$J_{(5,6)}$ = 3.16	H6$_{a/b}$	H6$_{a/b}$	H1,3,4,6$_{a}$	C5	77.72	H5	/
H6a	4.227	$J_{(6,6')}$ = 11.20	H5,6$_{b}$	H5,6$_{b}$	H5,6$_{b}$	C6	73.26	H6	/
H6b	4.136	$J_{(5,6')}$ = 8.68	H5,6$_{a}$	H5,6$_{a}$	H6a			H6′	

Table S2 ^{1}H/^{13}C chemical shifts of the oligosaccharides oAG-4, oAG-5, and oAG-6

**oAG-4**				**oAG-5-I**				**oAG-5-II**				**oAG-6**			
**A** β-D-Gal$_{4S6S}$-1,				**A** β-D-Gal$_{3S4S6S}$-1,				**A′** β-D-Gal$_{2S3S4S6S}$-1,				**A** β-D-Gal$_{2S3S4S6S}$-1,			
H1	4.675	C1	104.51	H1	4.663	C1	102.96	H1	4.732	C1	100.69	H1	4.757	C1	103.04
H2	4.386	C2	78.47	H2	4.211	C2	81.44	H2	4.355	C2	78.03	H2	4.323	C2	78.43
H3	4.453	C3	78.29	H3	3.880	C3	73.41	H3	4.491	C3	78.08	H3	4.450	C3	78.30
H4	4.984	C4	77.80	H4	4.691	C4	79.48	H4	4.989	C4	77.88	H4	4.981	C4	77.86
H5	4.003	C5	74.66	H5	3.960	C5	74.90	H5	4.017	C5	74.87	H5	4.017	C5	74.83
H6	4.199	C6	69.96	H6	4.196	C6	69.94	H6	4.226	C6	70.06	H6	4.206	C6	71.24
H6′	4.102			H6′	4.095			H6′	4.146			H6′	4.106		
**B** -2-D-2,3,4-tryhydroxylbutyric acid				**B -**4-D-GlcUANAc$_{6S}$				**B** -4-D-GlcUANAc				**B** -4-D-GlcUANAc$_{6S}$			
H1	/	C1	179.30	H1	/	C1	179.94	H1	/	C1	179.50	H1	/	C1	179.72
H2	4.109	C2	85.50	H2	4.168	C2	60.96	H2	4.208	C2	60.96	H2	4.182	C2	60.68
H3	3.916<br>3.648	C3	75.50	H3	4.333	C3	72.57	H3	4.383	C3	74.59	H3	4.312	C3	72.71
H4	3.620	C4	64.63	H4	3.921	C4	83.10	H4	4.283	C4	81.79	H4	3.933	C4	83.69
				H5	4.020	C5	72.03	H5	/	C5	178.51	H5	4.046	C5	72.28
				H6/6′	4.269<br>4.170	C6	71.23	H6	/	C6	176.54	H6/6′	4.238<br>4.181	C6	70.01
						C7	176.30	H7	2.545	C7	24.87				176.41
				H8	2.042	C8	24.84					H8	3.045		24.85
